# 6,6′-Biindeno[1,2-*b*]fluorene: an open-shell indenofluorene dimer†

**DOI:** 10.1039/d4sc03996c

**Published:** 2024-11-18

**Authors:** Himanshu Sharma, Palash Jana, Dibyendu Mallick, Subhajit Bandyopadhyay, Soumyajit Das

**Affiliations:** a Department of Chemistry, Indian Institute of Technology Ropar Rupnagar 140001 Punjab India chmsdas@iitrpr.ac.in; b Department of Chemical Sciences, Indian Institute of Science Education and Research (IISER) Kolkata Mohanpur 741246 West Bengal India; c Department of Chemistry, Presidency University Kolkata 700073 West Bengal India

## Abstract

Nakano *et al.* reported that the antiaromatic indenofluorene (IF) isomers are diradicaloid molecules having varying degrees of open-shell character, with indeno[1,2-*b*]fluorene displaying a weaker diradical character index (*y*_0_ = 0.072). Unlike 6,12-trimethylsilylethynyl disubstituted [1,2-*b*]IF, the 6,12-aryl disubstituted [1,2-*b*]IF derivatives did not show any experimental evidence of diradical properties. This raised the question of whether a [1,2-*b*]IF dimer would prefer a closed-shell or an open-shell ground state. To address this, herein we report the synthesis of a 6,6′-biindeno[1,2-*b*]fluorene derivative, which is a [1,2-*b*]IF dimer, constructed by linking two [1,2-*b*]IF units with a C–C single bond at carbons 6 and 6′ bearing the largest orbital coefficients for the highest occupied and lowest unoccupied molecular orbitals (HOMO and LUMO). The C6–C6′ linkage effectively narrowed the HOMO–LUMO gap while the strong desire to avoid *s*-indacene antiaromaticity restored two Clar sextets in two proaromatic *para*-quinodimethane subunits, resulting in an open-shell bifluorenylidene-type diradicaloid (*y*_0_ = 0.268) ground state with minor tetraradical character index (*y*_1_ = 0.007). The open-shell nature was confirmed by single crystal X-ray and electron paramagnetic resonance analyses, and supported by theoretical calculations.

## Abstract 2

Dimerization of antiaromatic indeno[1,2-*b*]fluorene units by linking the non-bridgehead carbons 6 and 6′ is reported to exhibit an open-shell ground state for the resulting biindenofluorene.

## Graphical abstract

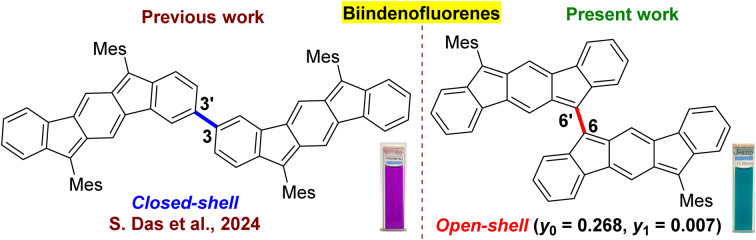

## Introduction

**Fig. 1:**
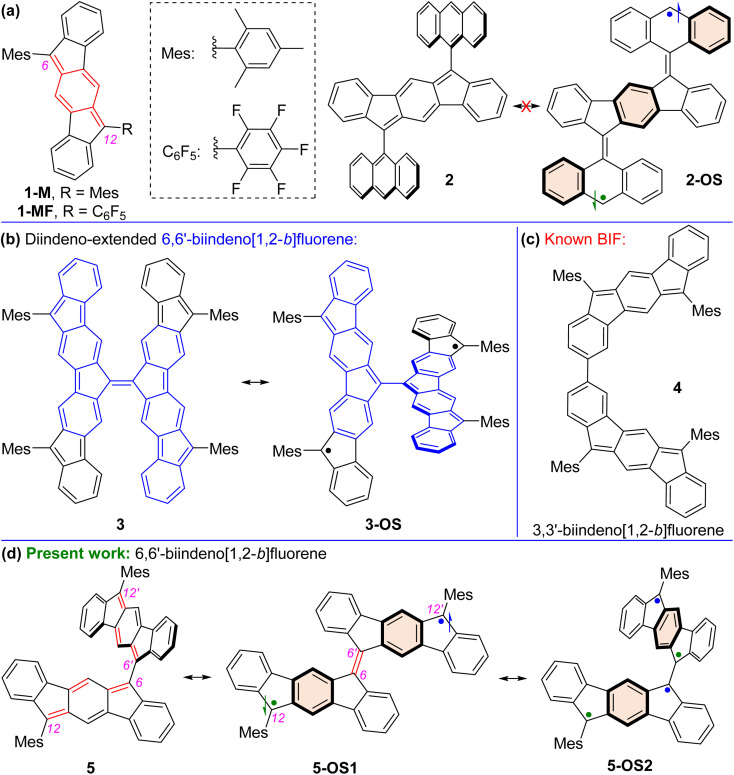
(a) Antiaromatic indeno[1,2-*b*]fluorene derivatives 1-M, 1-MF and 2; (b) Zimmerman and Stępień's tetrafluorenofulvalene (TFF) 3; (c) our reported 3,3′-biindeno[1,2-*b*]fluorene derivative 4; (d) closed-shell structure of targeted mesityl disubstituted 6,6′-biindeno[1,2-*b*]fluorene 5 and its representative open-shell (OS) diradicaloid bifluorenylidene-type structure 5-OS1 and tetraradicaloid biindenofluorene-type structure 5-OS2.

Open-shell (OS) singlet diradicaloid^1^ polycyclic hydrocarbons (PHs) have received widespread attention in the fields of organic synthesis,^2^ physical organic,^3^ structural organic,^4^ and organic materials^5^ chemistry. The recovery of aromaticity in proaromatic quinoidal PHs^6^ is a major driving force toward the OS ground state for higher order zethrenes,^7^ π-extended quinodimethanes (QDMs),^8^ anthenes,^9^ benzenoid^10^ and non-benzenoid acenes,^11^*peri*-acenoacenes,^12^ and formally antiaromatic indenofluorene (IF) isomers.^13^ The five IF isomers are predicted to be OS diradicaloids,^13*c*,13*f*^ but the degree of diradical character (*y*_0_) for indeno[1,2-*a*]fluorene and indeno[2,1-*b*]fluorene is larger than that of the other three isomers including indeno[1,2-*b*]fluorene (*y*_0_ = 0.072). Indeed, 6,12-aryl and 6,12-triisopropylsilylethynyl (TIPSE) disubstituted [1,2-*b*]IFs^14^ didn't show any experimental evidence of OS diradicaloid features despite carrying a proaromatic *para*-QDM (*p*-QDM shown in red, Fig. 1a) subunit in the π-backbone. Antiaromatic molecules are regarded as delocalized diradicals.^15^ Zhao reported that replacing bulkier TIPSE with a trimethylsilylethynyl group in [1,2-*b*]IF resulted in cyclo-oligomeric products due to diradical contribution of the [1,2-*b*]IF,^16^ clearly indicating its OS character.^13*c*,17^

Aryl substitutions on the apical carbons (C6 and C12, Fig. 1a) of [1,2-*b*]IF for either symmetrical^14*b*^1-M or unsymmetrical^18^1-MF didn't allow any diradical properties to be observed experimentally, despite lowering of the highest occupied molecular orbital (HOMO)-lowest unoccupied molecular orbital (LUMO) energy gap (hereafter denoted as HLG) in 1-MF than 1-M due to a smaller dihedral angle between the C_6_F_5_ unit and IF backbone enabling a greater π-delocalization. Quinoidal π-extension of the *s*-indacene core in the form of 2,6-anthraquinodimethane (2,6-AQDM) may result in an OS π-extended [1,2-*b*]IF,^19^ similar to non-benzenoid bis(phenalenyl)^20^ and benzenoid nonazethrene^7*b*^ bearing a 2,6-AQDM core. While the 2,6-naphthaquinodimethane (2,6-NQDM)-embedded π-extended [1,2-*b*]IF was originally reported to be closed-shell (CS), its OS properties were accessed subsequently only above 200 °C.^21^ Notably, benzenoid octazethrene containing a 2,6-NQDM subunit is an OS molecule.^22^

Alongside aromaticity recovery, a small HLG is crucial^23^ for PHs displaying OS ground state. Extension of the π-delocalization path is the key to decrease the HLG. In this regard, Yamashita's work on anthryl disubstituted [1,2-*b*]IF 2 is noteworthy (Fig. 1a).^24^ Molecule 2 displayed a CS ground state, though one can draw structure 2-OS with three additional aromatic benzene rings (Clar sextets^7*d*^). Presumably, the large dihedral angle (∼76°) between the anthryl groups and IF backbone for 2 inhibits efficient π-delocalization, and thus recovery of the three Clar sextets in 2-OS has negligible contribution to the ground state structure. Sterically overcrowded alkene bridged PHs have lately gained attention as novel OS diradicaloids as they release steric strain and recover Clar sextets when switching from the Kekulé-type CS quinoidal form to OS diradical form.^25^ Tetrafluorenofulvalene (TFF)^26^3 is the latest example among them bearing two π-extended [1,2-*b*]IF units connected through an apical olefinic C–C linkage (Fig. 1b). TFF 3 was viewed^26^ as a diradicaloid 3-OS bearing diindeno-fused 6,6′-biindeno[1,2-*b*]fluorene (6,6′-[1,2-*b*]BIF unit shown in blue for 3). While 3 clearly exhibited an OS singlet ground state with a small singlet(S)–triplet(T) energy gap (Δ*E*_S–T_) due to the release of steric strain and recovery of Clar sextets as it switches from its non-aromatic olefinic form to polyradical forms, what remained unknown is whether the 6,6′-[1,2-*b*]BIF motif without diindeno-radical (shown in black for 3-OS) fusion exhibits OS properties.

We envisaged the design of 12,12′-dimesityl-6,6′-biindeno[1,2-*b*]fluorene 5 that can recover two additional Clar sextets in its OS diradical 5-OS1 and tetraradical 5-OS2 forms (Fig. 1d). The 5-OS2 and 5 forms are viewed as biindenofluorene (BIF) motifs while 5-OS1 is a diindeno-fused bifluorenylidene (BF) diradical. BF usually prefers a twisted form over the folded form,^25*a*^ and BF-based materials are promising hole and electron transporters in organic photovoltaics.^27^ Interestingly, an extended quinoidal form of 6,6′-[1,2-*b*]BIF was theoretically shown to contribute to a poly-IF which is a π-elongated BF-type structure.^28^ However, synthesis of a hypothetical smaller quinoidal fragment like 5, which majorly contributes to the ground state properties of Scherf's poly-IF,^28^ remained unknown thus far. Our recently reported BIF 4 (Fig. 1c) displayed a CS ground state, which was formed by dimerizing two [1,2-*b*]IF units through carbons 3 and 3′ with appreciable HOMO and LUMO coefficients.^29^ Because the apical carbons 6/6′(12) contain larger HOMO and LUMO distributions than those of carbons 3/3′(9),^29^ we hypothesized that dimerizing two [1,2-*b*]IF units by apical carbons 6 and 6′ may significantly influence both HOMO and LUMO energy levels in 5.

Moreover, the large alternation of C=C/C–C bonds of the *p*-QDM subunit of *s*-indacene makes 5 a polyene-like system considering the apical π-conjugation path resembling a dodecahexaene subunit (shown in red for 5, Fig. 1d). This polyene subunit may act as a plausible π-delocalization path due to a smaller inter-IF torsional angle. It is known that the C–C double bond for ethylene is quite short, while in longer polyene chain, an increase of the C–C double bond and shortening of the adjacent C–C single bond may occur, leading to an extension of π-conjugation and a decrease of HLG.^30^ A small torsional angle may lead to a greater electronic communication between the IF units for 5, resulting in a smaller HLG. The hypotheses of a smaller HLG for the BIF 5 than that of 4 and recovery of two Clar sextets in OS forms for 5 enthused us to explore the ground state properties of 5 by computational and experimental approaches.

## Results and discussion

**Fig. 2:**
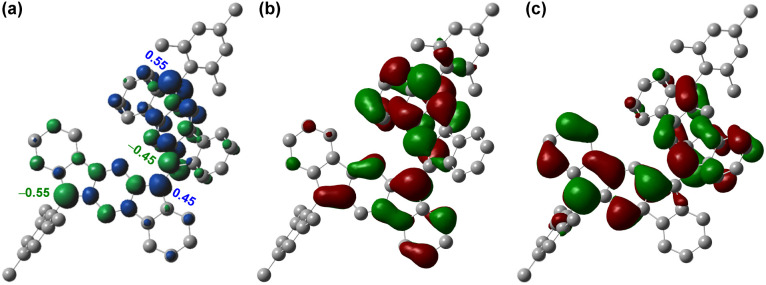
(a) Spin density map of 5. Isovalue for surfaces: MO = 0.02, density = 0.005; frontier molecular orbital profiles for the (b) α-spin and (c) β-spin of 5.

Since a *para*-quinoidal 6,6′-[1,2-*b*]BIF-type π-extended structure is reported to dominate the ground state of BF-type poly-IF to favor mutual distortion,^28^ we first conducted density functional theory (DFT) calculations to examine if 5 is OS in the ground state. The DFT calculations of 5, using the same level of theory ((U)CAM-B3LYP/6-31G(d,p)) as that used for 3,^26^ suggested that the energy of the OS singlet state is 4.85 kcal mol^−1^ lower than that of the triplet state (*i.e.* Δ*E*_S–T_ = −4.85 kcal mol^−1^, Table S1†). The frontier molecular orbital (FMO) profiles of singly occupied molecular orbitals (SOMO) display a characteristic disjointed nature of the alpha (SOMO-α) and beta (SOMO-β) spins for the ground state singlet diradical (Fig. 2b and c), while the spin densities are found to be delocalized over the π-conjugated IF backbone with four apical carbons bearing the larger spin densities (Fig. 2a). The large spin densities on the four apical carbons imply singlet diradical and tetraradical characters, which were found to be 26.8% and 0.7%, respectively, based on the natural orbital occupancy number (NOON) calculations (see ESI†).

The negligible tetraradical character of 5 may be attributed to the minor driving force (no additional Clar sextet recovery; only a minor inter-IF steric clash) toward 5-OS2 from 5-OS1 (Fig. 1d). However, the strong desire^6*a*^ to avoid *s*-indacene antiaromaticity through recovery of two Clar sextets may drive quinoidal 5 to become diradicaloid 5-OS1 in the singlet ground state by enhancing the C6–C6′ π-bond character while locating radicals at apical carbons 12 and 12′ bearing the largest spin densities (Mulliken spin density of 0.55). As molecules with intermediate diradical character are important for non-linear optics, singlet fission and molecular electronics,^3,5^ we designed a sterically promoted synthetic approach to construct 5 and study its ground state characteristics experimentally.

**Scheme 1:**
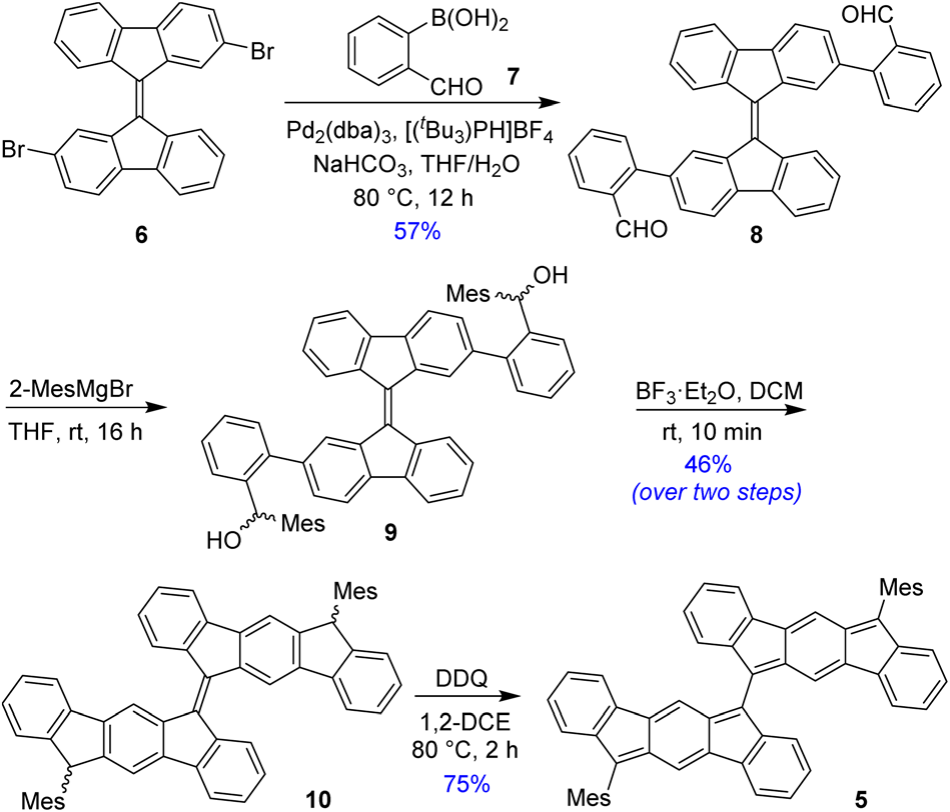
Synthesis of BIF 5.

**Fig. 3:**
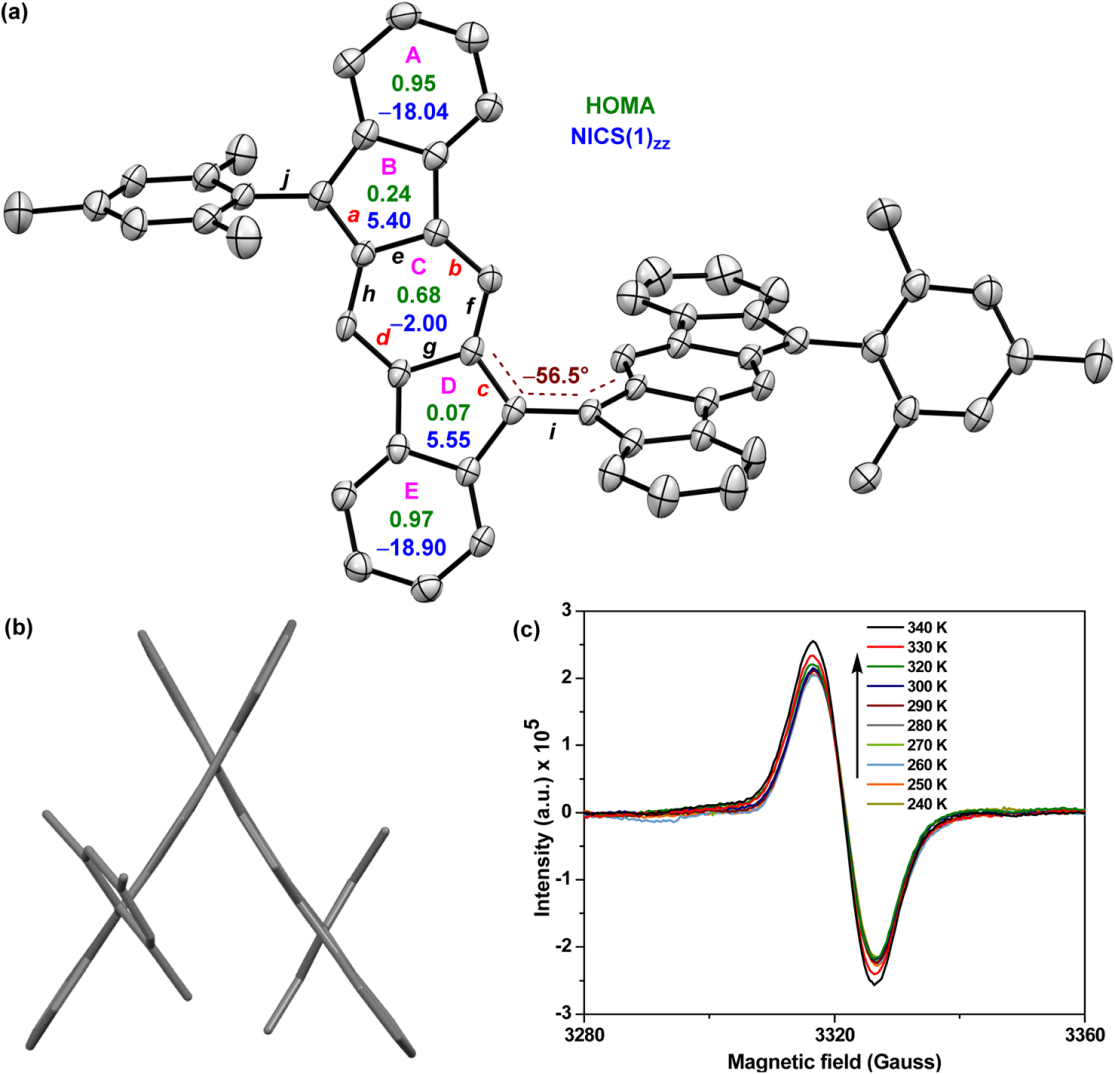
(a) X-ray crystallographic structure of 5 with the ellipsoids drawn at 30% probability level (hydrogens omitted), including NICS(1)_*zz*_ (blue) and HOMA (green) indices; (b) side-view of 5; (c) EPR spectra of 5 recorded at variable temperatures for the powder form.

BIF 5 was synthesized in multiple steps as illustrated in Scheme 1. 2,2′-Dibromo-9,9′-bifluorenylidene 6 was synthesized as a diastereomeric mixture following a literature method^31^ (Fig. S1†), and then it was subjected to a two-fold Suzuki coupling with (2-formylphenyl)boronic acid to afford dialdehyde 8 in 57% yield. Treatment of 8 with an excess of 2-mesitylmagnesium bromide at room temperature gave dicarbinol 9, which, without purification, was treated with BF_3_·Et_2_O in dichloromethane (DCM) to afford dihydro derivative 10 (46% yield over two steps) by utilizing the steric crowding of bulky mesityl (Mes) groups.^18^ Treatment of dihydro precursor 10 with 2,3-dichloro-5,6-dicyano-1,4-benzoquinone (DDQ) in 1,2-dichloroethane (1,2-DCE) for 2 h at 80 °C produced 5 as a blue solid in 75% yield after silica gel column chromatographic purification. While nuclear magnetic resonance (NMR, see ESI†) and high-resolution mass spectrometry (HRMS) analyses indicated the formation of desired product 5, unambiguous structural confirmation was obtained from single crystal X-ray diffractometry (SCXRD) analysis (Fig. 3a).

Single crystals^32^ of 5 were obtained by slow solvent diffusion of methanol into DCM/carbon disulfide (1 : 1) solution. As depicted in Fig. 3a and b, the two [1,2-*b*]IF units of twisted BIF 5 form a torsional angle of ∼56.5° which is much smaller than that of anthryl disubstituted [1,2-*b*]IF 2,^24^ suggesting a greater π-delocalization/electronic communication between two IF units. The bond lengths (bonds *a* to *j* are labelled in Fig. 3a) of 5 from SCXRD analyses are in line with those from DFT analyses, and are summarized in Table 1. The π-delocalization is evident from the C–C bond length analyses (Table 1; see the ESI† for full bond length analyses including e.s.d values), as an increase of the C–C double bond lengths (C_sp^2^_=C_sp^2^_ bonds *a*, *b*, *c*, *d*) for the *p*-QDM subunit of 5 was clearly observed when compared to those of symmetrical 1-M (*a*/*c* and *b*/*d*) and unsymmetrical 1-MF. At the same time, the C–C single bond lengths (C_sp^2^_–C_sp^2^_ bonds *e*, *f*, *g*, *h*) of the *p*-QDM subunit of 5 were also found to be shorter than those of the 1-M (*e*/*g* and *f*/*h*). Moreover, the bond length for central C–C single bond *i* connecting the IF units for 5 was found to be 1.452 Å (DFT: 1.414 Å), which corresponds to a formal single bond in the CS configuration. However, it is shorter than the C–C single bond *j* (crystal: 1.501 Å; DFT: 1.480 Å) connecting the mesityl group and [1,2-*b*]IF core, and clearly shorter than the C–C single bond connecting the anthryl and [1,2-*b*]IF rings in 2 (1.492 Å).^24^ The distance is comparable to BIF 4 (1.451 Å),^29^ suggesting some π-bond character for the central C–C bond *i* in 5 which is strengthened due to π-electron delocalization.

**Table 1:** Comparison of mean C–C bond lengths (Å) for 5, 1-MF, and 1-M

Bonds^a^	5^b^	5^c^	1-MF^d^	1-M^e^
*a*	1.392	1.403	1.372	1.380
*b*	1.364	1.368	1.349	1.356
*c*	1.392	1.429	1.373	1.380
*d*	1.367	1.371	1.346	1.356
*e*	1.456	1.441	1.433	1.467
*f*	1.415	1.416	1.456	1.433
*g*	1.459	1.436	1.435	1.467
*h*	1.416	1.414	1.457	1.433
*i*	1.452	1.414	—	—
*j*	1.501	1.480	1.482	1.484

a *a*–*j* bonds are labelled in Fig. 3a.

b SCXRD data.

c DFT data.

d SCXRD data from ref. 18.

e SCXRD data from ref. 14b.

The above findings clearly indicate the major contribution of a diradicaloid structure 5-OS1 in the electronic ground state of 5, which is in line with the computational analyses and the broad NMR signals observed for core protons due to thermally populated triplet species (Fig. S8†). Electron paramagnetic resonance (EPR) studies of solid 5 displayed a featureless broad EPR signal (Fig. 3c), and when the temperature dropped, the signal intensity dropped as well. This can be explained by the fact that triplet biradical species have smaller populations at lower temperatures, thus further confirming the OS singlet ground state for 5 with Δ*E*_S–T_ = −4.35 ± 0.6 kcal mol^−1^, as obtained by fitting of the EPR data using the Bleaney–Bowers equation (Fig. S14†).^23*b*,33^

**Fig. 4:**
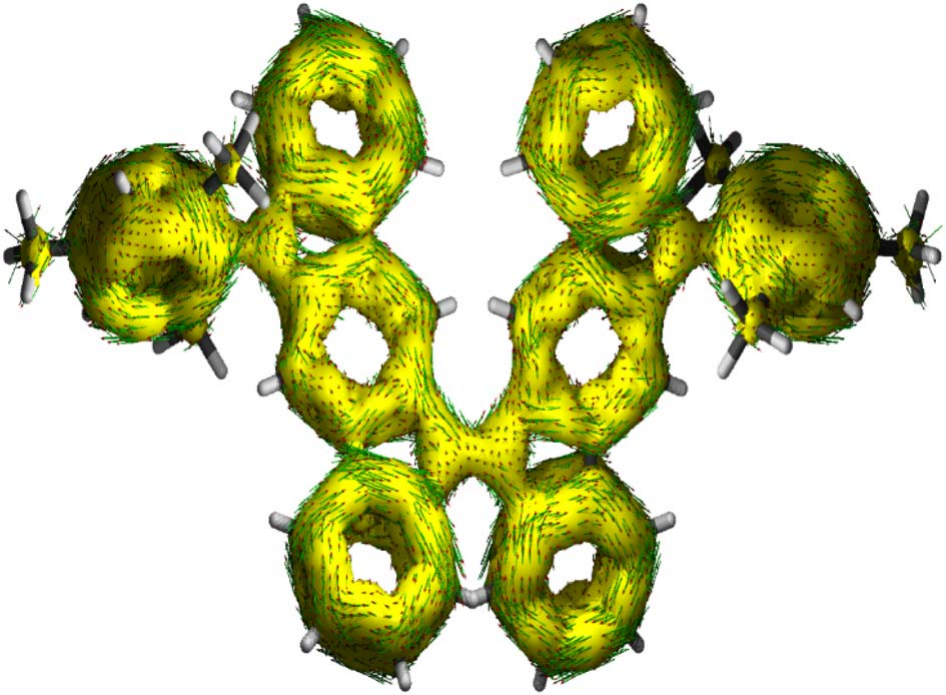
Current–density vectors plotted onto the ACID isosurface of 0.02 for the π system of 5.

The harmonic oscillator model of aromaticity (HOMA, Fig. 3a)^34^ for the ground state structure of 5 suggested insignificant bond length alternation (BLA) for rings A (0.95) and E (0.97) and large BLA for rings B (0.24) and D (0.07), similar to 1-MF.^18^ However, a not so large BLA was found for the benzenoid ring C (0.68), implying its moderate aromatic character according to the geometrical criterion of aromaticity. Nucleus independent chemical shift [NICS(1)_*zz*_, Fig. 3a]^35^ calculations for the ground state structure of 5 using the BHandHLYP functional^36^ suggested aromaticity for rings A (−18.04) and E (−18.90) and moderate antiaromaticity for rings B (5.40) and D (5.55). A weak aromatic character for the central benzenoid ring C (−2.0) for 5, which is in line with the HOMA analysis, further indicated a major contribution of the OS diradical form to the electronic ground state. To support the NICS analysis, we conducted anisotropy of the induced current density^37^ (ACID) calculation for 5 and current density vectors were plotted onto the ACID isosurfaces (Fig. 4). The ACID plot clearly exhibited strong diatropicity (clockwise ring current) for rings A and E, and a diatropic ring current with low intensity over ring C, signifying strong to weak aromaticity for six-membered rings A/E to C. The five-membered rings B and D were found to be atropic (nonaromatic), implying the diatropic ring currents of fused benzene rings could induce some paratropic effect.^38^ The aromaticity indices overall suggested that the contribution of the diradical structure 5-OS1 is much more important for 5 in the singlet ground state.

**Fig. 5:**
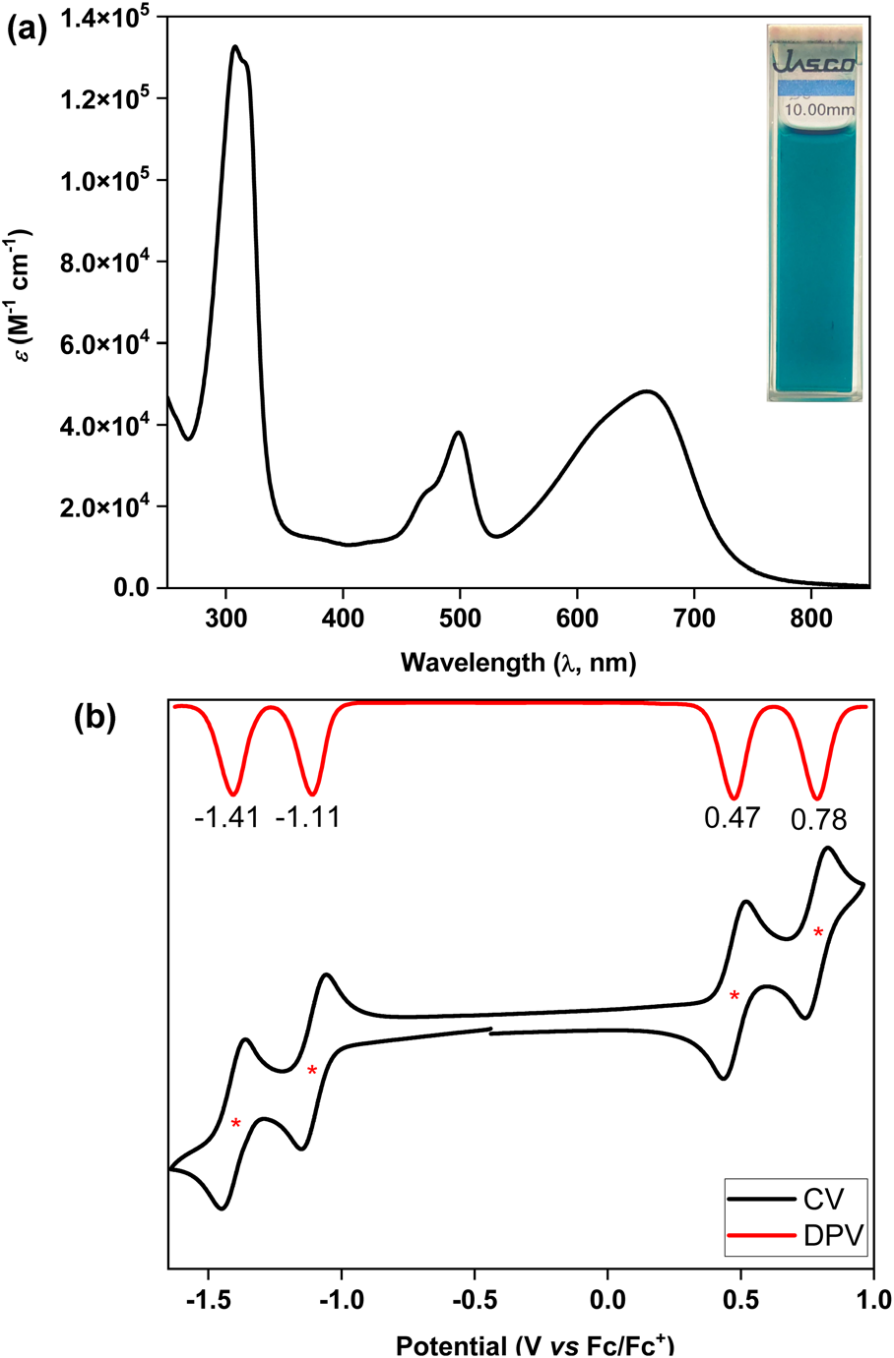
(a) UV-vis-NIR spectrum of 5 in chloroform; (b) CV and DPV of 5.

The ultraviolet-visible-near infrared (UV-vis-NIR) spectrum (Fig. 5a) of teal colored 5 in chloroform displayed a broad absorption band in the low energy region at *λ*_max_ = 661 nm (*ε* = 48 000 M^−1^ cm^−1^), which may be attributed to the HOMO → LUMO transition according to time dependent-DFT (TD-DFT: *λ*_max_(TD) = 582 nm, oscillator strength (*f*) = 0.7125) calculations (Table S3†), with the absorption tail extended to ∼825 nm in the NIR region. The lowest energy band for 5 was found to be 98 nm red-shifted than that of 4, implying a greater electronic communication in 5, which is also reflected by an enhancement of the molar extinction coefficient (*ε*). The optical HLG of 5 is 1.50 eV, as roughly estimated from the absorption onset, which is 0.10 eV and 0.52 eV smaller than those of its BIF 4 and bipentacene (BP)^39^ counterparts, respectively. BIF 5 was found to be non-emissive by the naked eye, similar to the monomer,^40^ and quite stable under ambient conditions with a half-life of 19 days in toluene (Fig. S13†).

Cyclic voltammetry (CV) and differential pulse voltammetry (DPV) analyses of 5 in the DCM/Bu_4_NPF_6_ solvent/electrolyte couple exhibited four-stage redox amphotericity (Fig. 5b), displaying two reversible reduction waves with half-wave potentials *E*^red1^_1/2 _= −1.11 V and *E*^red2^_1/2 _= −1.41 V and two reversible oxidation waves at *E*^ox1^_1/2_ = 0.47 V and *E*^ox2^_1/2 _= 0.78 V (*vs.* ferrocene/ferrocenium (Fc/Fc^+^)). The excellent reversibility of the redox waves for 5 may be attributed to the dominance of an OS ground state, suggesting promise for application in organic electronics.^5*c*,41^ The HOMO and LUMO energy levels of 5 were found to be −5.18 and −3.80 eV, respectively, based on the onset redox potentials, affording an electrochemical HLG of 1.38 eV, which is 0.36 eV smaller than that of 4. Notably, the low-lying LUMO of BIF 5 is remarkably stabilized by 0.82 eV when compared to that of its aromatic BP counterpart with the same solubilizing group.^39^

## Conclusions

In summary, we have designed and synthesized a stable diradicaloid BIF 5, as demonstrated by computational and experimental analyses, that exhibits four-stage redox amphotericity and improved optoelectronic properties compared to the known BIF and BP counterparts.^29,39^ Our BIF gains a sterically hindered BF-type OS diradicaloid ground state through gaining of partial double bond character for the inter-IF C–C single bond, which, by design, is opposite to the conventionally designed sterically overcrowded alkene-type switchable di/tetra-radicaloids.^25,26^ A shorter C_sp^2^_–C_sp^2^_ single bond length linking the two [1,2-*b*]IF units was found in the SCXRD analysis of 5, which is attributed to the better π-electron delocalization between two IF cores owing to a reduced inter-IF torsional angle as they are π-extended through carbons 6 and 6′ of [1,2-*b*]IF units bearing large HOMO and LUMO distributions. Our study showed that the oligomerization of antiaromatic IF units through appropriate carbon positions may significantly influence the optoelectronic properties, as the electronic ground state was effectively tuned. Syntheses of other regioisomeric BIF diradicaloids for organic electronics and photonics studies are presently underway.

## Data availability

The data that support the findings of this study are available in the ESI† of this article.

## Author contributions

H. S. performed the synthesis and characterization of all compounds. S. D. and H. S. performed DFT calculations. P. J. and S. B. performed EPR measurements. D. M. performed ACID calculations. S. D. conceived the idea, supervised the project, secured funding, and wrote the manuscript together with H. S. All authors gave approval to the final version of the manuscript.

## Conflicts of interest

There are no conflicts to declare.

## Supplementary Material

SC-OLF-D4SC03996C-s001

SC-OLF-D4SC03996C-s002

## References

[cit1] Wu J. , Diradicaloids, Jenny Stanford Publishing, New York, 2022

[cit2] Hu J., Xiang Q., Tian X., Ye L., Wang Y., Ni Y., Chen X., Liu Y., Chen G., Sun Z. (2024). J. Am. Chem. Soc..

[cit3] Muhammad S., Nakano M., Al-Sehemi A. G., Kitagawa Y., Irfan A., Chaudhry A. R., Kishi R., Ito S., Yoneda K., Fukuda K. (2016). Nanoscale.

[cit4] Liu C., Sandoval-Salinas M. E., Hong Y., Gopalakrishna T. Y., Phan H., Aratani N., Herng T. S., Ding J., Yamada H., Kim D., Casanova D., Wu J. (2018). Chem.

[cit5] Jousselin-Oba T., Mamada M., Marrot J., Maignan A., Adachi C., Yassar A., Frigoli M. (2019). J. Am. Chem. Soc..

[cit6] Zeng Z., Shi X., Chi C., Navarrete J. T. L., Casado J., Wu J. (2015). Chem. Soc. Rev..

[cit7] Sun Z., Zeng Z., Wu J. (2014). Acc. Chem. Res..

[cit8] Zeng Z., Sung Y. M., Bao N., Tan D., Lee R., Zafra J. L., Lee B. S., Ishida M., Ding J., Navarrete J. T. L., Li Y., Zeng W., Kim D., Huang K., Webster R. D., Casado J., Wu J. (2012). J. Am. Chem. Soc..

[cit9] Konishi A., Hirao Y., Nakano M., Shimizu A., Botek E., Champagne B., Shiomi D., Sato K., Takui T., Matsumoto K., Kurata H., Kubo T. (2010). J. Am. Chem. Soc..

[cit10] Bendikov M., Duong H. M., Starkey K., Houk K. N., Carter E. A., Wudl F. (2004). J. Am. Chem. Soc..

[cit11] Maekawa T., Ueno H., Segawa Y., Haley M. M., Itami K. (2016). Chem. Sci..

[cit12] Jousselin-Oba T., Mamada M., Wright K., Marrot J., Adachi C., Yassar A., Frigoli M. (2022). Angew. Chem., Int. Ed..

[cit13] Shimizu A., Kishi R., Nakano M., Shiomi D., Sato K., Takui T., Hisaki I., Miyata M., Tobe Y. (2013). Angew. Chem., Int. Ed..

[cit14] Chase D. T., Rose B. D., McClintock S. P., Zakharov L. N., Haley M. M. (2011). Angew. Chem., Int. Ed..

[cit15] Abe M. (2013). Chem. Rev..

[cit16] Fu X., Zhao D. (2015). Org. Lett..

[cit17] Moles Quintero S., Haley M. M., Kertesz M., Casado J. (2022). Angew. Chem., Int. Ed..

[cit18] Sharma H., Bhardwaj N., Das S. (2022). Org. Biomol. Chem..

[cit19] Rudebusch G. E., Zafra J. L., Jorner K., Fukuda K., Marshall J. L., Arrechea-Marcos I., Espejo G. L., Ortiz R. P., Gómez-García C. J., Zakharov L. N., Nakano M., Ottosson H., Casado J., Haley M. M. (2016). Nat. Chem..

[cit20] Shimizu A., Hirao Y., Matsumoto K., Kurata H., Kubo T., Uruichib M., Yakushib K. (2012). Chem. Commun..

[cit21] Barker J. E., Frederickson C. K., Jones M. H., Zakharov L. N., Haley M. M. (2017). Org. Lett..

[cit22] Li Y., Heng W., Lee B. S., Aratani N., Zafra J. L., Bao N., Lee R., Sung Y. M., Sun Z., Huang K., Webster R. D., Navarrete J. T. L., Kim D., Osuka A., Casado J., Ding J., Wu J. (2012). J. Am. Chem. Soc..

[cit23] Zeng W., Qi Q., Wu J. (2018). Eur. J. Org Chem..

[cit24] Nishida J.-i., Tsukaguchi S., Yamashita Y. (2012). Chem.–Eur. J..

[cit25] Ishigaki Y., Harimoto T., Shimajiri T., Suzuki T. (2023). Chem. Rev..

[cit26] Prajapati B., Ambhore M. D., Dang D., Chmielewski P. J., Lis T., Gómez-García C. J., Zimmerman P. M., Stępień M. (2023). Nat. Chem..

[cit27] Rakstys K., Saliba M., Gao P., Gratia P., Kamarauskas E., Paek S., Jankauskas V., Nazeeruddin M. K. (2016). Angew. Chem., Int. Ed..

[cit28] Reisch H., Wiesler U., Scherf U., Tuytuylkov N. (1996). Macromolecules.

[cit29] Sharma H., Ankita, Mittal V., Pandey U. K., Das S. (2024). Org. Lett..

[cit30] Suaud N., Amor N. B., Guihéry N., Malrieu J. (2021). Theor. Chem. Acc..

[cit31] Xu J., Takai A., Bannaron A., Nakagawa T., Matsuo Y., Sugimoto M., Matsushita Y., Takeuchi M. (2018). Mater. Chem. Front..

[cit32] CCDC 2362686† for 5

[cit33] Jana P., Koppayithodi S., Mahato S., Molla S., Bandyopadhyay S. (2023). J. Phys. Chem. Lett..

[cit34] Lu T., Chen F. (2012). J. Comput. Chem..

[cit35] Fallah-Bagher-Shaidaei H., Wannere C. S., Corminboeuf C., Puchta R., Schleyer P. v. R. (2006). Org. Lett..

[cit36] Lehtola S., Dimitrova M., Fliegl H., Sundholm D. (2021). J. Chem. Theory Comput..

[cit37] Geuenich D., Hess K., Köhler F., Herges R. (2005). Chem. Rev..

[cit38] Sharma P. K., Babbar A., Mallick D., Das S. (2023). J. Org. Chem..

[cit39] Lukman S., Musser A. J., Chen K., Athanasopoulos S., Yong C. K., Zeng Z., Ye Q., Chi C., Hodgkiss J. M., Wu J. (2015). Adv. Funct. Mater..

[cit40] Rose B. D., Shoer L. E., Wasielewski M. R., Haley M. M. (2014). Chem. Phys. Lett..

[cit41] Gopalakrishna T. Y., Zeng W., Lua X., Wu J. (2018). Chem. Commun..

