# Supplementary material for: 6,6′-Biindeno[1,2-b]fluorene: an open-shell indenofluorene dimer
Source: Chem Sci. 2024 Nov 18;15(48):20215–22. doi: 10.1039/d4sc03996c (PMC11587529; doi:10.1039/d4sc03996c)
Supplement: SC-015-D4SC03996C-s001 [file SC-015-D4SC03996C-s001.pdf]

Supplementary Information (SI) for Chemical Science.
This journal is © The Royal Society of Chemistry 2024

Electronic Supplementary Information (ESI)

6,6'-Biindeno[1,2-*b*]fluorene: an open-shell indenofluorene dimer

Himanshu Sharma,[a] Palash Jana,[b] Dibyendu Mallick,[c] Subhajit Bandyopadhyay,[b] and Soumyajit Das*[a]

[a]Department of Chemistry, Indian Institute of Technology Ropar, Rupnagar 140001, Punjab, India, E-mail: chmsdas@iitrpr.ac.in

[b]Department of Chemical Sciences, Indian Institute of Science Education and Research (IISER) Kolkata, Mohanpur 741246, West Bengal, India.

[c]Department of Chemistry, Presidency University, Kolkata 700073, West Bengal, India.

Table of Contents

1. Experimental Section………………………………………………………………. S2
   1.1 General Information………………………………………………………… S2
   1.2 Synthetic procedures and characterization data………………………………... S3
   1.3 NMR Spectra……………………………………………………………… S5
2. DFT Calculations……………………………………………………………... S10
3. X-ray crystallographic analyses………………………………………………… S12
4. Photostability test…………………………………………………………….. S14
5. EPR measurement and Bleaney-Bowers plot……………………………………. S15
6. Appendix………………………………………………………………………. S16
7. References…………………………………………………………………..... S25

1. Experimental section

<u>1.1 General information</u>: Chemicals and reagents were purchased from commercial suppliers (Merck, GLR innovations, BLD pharm, Spectrochem) and used without further purification. Thin layer chromatography (TLC) was performed using pre-coated silica-plates purchased from Merck (silica gel 60 PF254, 0.25 mm). Column chromatography was performed using silica gel 100-200 mesh. NMR spectra were recorded in $CDCl_3$ at room temperature, on JEOL JNM-ECS400 spectrometer at operating frequencies of 400 MHz (^{1}H) or 100 MHz (^{13}C) as indicated in the individual spectrum. Chemical shifts (δ) are given in ppm relative to residual solvent (chloroform δ = 7.26, 1,1,2,2-tetrachloroethane δ = 6.00 ^{1}H, and δ = 77.16 for proton-decoupled ^{13}C NMR), and coupling constants (*J*) are expressed in hertz (Hz). Multiplicity is tabulated as s for singlet, d for doublet, dd for doublet of doublet, t for triplet, q for quartet and m for multiplet. Structural assignments were made with additional information from gCOSY, and gNOESY experiments. High-resolution mass spectra (HRMS) were recorded using electronspray ionization (ESI) methods on Waters (XEVO G2-XS QTOF) mass spectrometer. UV-vis-NIR spectra were recorded in JASCO V-770 spectrophotometer. Cyclic voltammetry measurements of **5** (0.001 M) was performed using an electrochemical analyzer potentiostat model CHI-1115C from CH Instruments with a conventional three-electrode cell at 298 K under a nitrogen atmosphere at a scan rate of 50 mV/s. This electrochemical cell contains a platinum (Pt) disk (disk shaped with 2-mm diameter) as a working electrode, Pt wire as a counter electrode, and silver (Ag) wire as a pseudo-reference electrode. The Pt working electrode was polished with 1.0 micron α-alumina polishing powder in a figure-eight motion. Electrolyte solution (0.1 M) was prepared by dissolving tetrabutylammonium hexafluorophosphate (Bu_4NPF_6) in 5 mL dry dichloromethane (DCM). The DCM solution was degassed by bubbling nitrogen gas for 10 minutes prior to measurements. The direction of initial scan was chosen as reductive, with the initial potential values set at zero for **5** and switching between negative potential (− 1.2 V) first, and then going to positive potential (1.4 V), and finally returning to zero (vs Ag wire). The potential was externally calibrated against the ferrocene/ferrocenium (Fc/Fc^+) couple (0.43 V). Melting points were determined using Cole-Parmer MP 250D-P melting point analyzer. X-band electron paramagnetic resonance (EPR) spectra were recorded using an EMX MICRO X Bruker EPR instrument.

1.2 Syntheses and characterization data

2,2'-([9,9'-bifluorenylidene]-2,2'-diyl)dibenzaldehyde (8): An oven-dried thick-walled glass tube was charged with pre-synthesized **6** (800 mg, 1.65 mmol), (2-formylphenyl)boronic acid (987 mg, 6.58 mmol) **7**, $NaHCO_3$ (2.76 g, 32.91 mmol), tetrahydrofuran (8 mL), distilled water (0.6 mL) and purged with N_2 for 30 mins. $Pd_2(dba)_3$ (151 mg, 0.164 mmol), and [(*t*-Bu)$_3$PH]BF_4 (191 mg, 0.658 mmol) were added subsequently and the resulting mixture was heated at 80 °C using an oil bath for 12 h. After cooling to room temperature, the THF was evaporated, water was added and the resultant mixture was extracted with DCM (3 x 50 mL). The organic phase was dried over Na_2SO_4, and evaporated under reduced pressure. The residue was subjected to column chromatography (SiO_2, DCM/hexane 70:100) to give dialdehyde **8** as orange solid. (507 mg, 57% yield): R_f = 0.46 (20% EtOAc/hexanes); mp = 165-166°C; ^{1}H NMR (400 MHz, $CDCl_3$) δ 10.06 (m, 2H), 8.50 – 8.45 (m, 1H), 8.38 – 8.35 (m, 2H), 8.01 (td, *J* = 7.9, 1.6 Hz, 2H), 7.80 – 7.73 (m, 4H), 7.62 (td, *J* = 7.4, 1.4 Hz, 1H), 7.50 – 7.44 (m, 4H), 7.43 – 7.34 (m, 4H), 7.30 – 7.24 (m, 3H), 7.12 (d, *J* = 7.4 Hz, 1H); ^{13}C{^{1}H} NMR (100 MHz, $CDCl_3$) δ 192.2, 192.1, 146.0, 145.8, 141.2, 140.8, 138.4, 138.4, 136.6, 136.4, 133.9, 133.8, 133.7, 131.6, 131.2, 130.8, 130.5, 129.8, 128.1, 128.0, 127.9, 127.7, 127.6, 126.9, 126.8, 120.4, 120.1, 119.7; HRMS (ESI) *m/z*: $[M + H]^+$ calcd for $C_{40}H_{25}O_2$ 537.1855, found 537.1854 (error: -0.2 ppm).

12,12'-dimesityl-12H,12'H-6,6'-biindeno[1,2-*b*]fluorenylidene (10): To a dry THF solution (10 ml) of dialdehyde **8** (285 mg, 0.531 mmol), was added 2-mesitylmagnesiumbromide (2.66 ml, 2.66 mmol, 5 equiv) at room temperature under N_2 atmosphere, and the resulting solution was stirred for 16 h. After consumption of starting material (monitored by TLC), saturated ammonium chloride was added, the organic phase was separated, and the aqueous phase was extracted with DCM. The combined organic phase was washed with brine, dried over Na_2SO_4, and concentrated under reduced pressure to give crude alcohol **9**. Without further purification,

the crude dicarbinol **9** was subjected to Friedel-Craft cyclization reaction with $BF_3{\cdot}OEt_2$ (0.1 ml) in a DCM solution. After stirring the reaction mixture for 10 min at room temperature, a saturated aqueous NH_4Cl solution was added to the reaction mixture and it was extracted with DCM (3 x 100 mL). The organic layer was dried over anhydrous Na_2SO_4, filtered, and removed under reduced pressure to afford a residue which was purified by silica gel column chromatography (EtOAc/hexane 2:100) to give **10** as orange-reddish solid. (180 mg, 46% yield in two steps): R_f = 0.42 (5% EtOAc/hexanes); mp = 251-252°C; ^{1}H NMR (400 MHz, $CDCl_3$) δ 9.05 (d, *J* = 4.1 Hz, 1H), 8.87 (s, 1H), 8.53 (d, *J* = 7.8 Hz, 1H), 8.40 (d, *J* = 7.8 Hz, 1H), 7.72 (d, *J* = 7.5 Hz, 1H), 7.65 – 7.55 (m, 5H), 7.36 – 7.28 (m, 4H), 7.24 – 7.16 (m, 7H), 7.10 (t, *J* = 2.7 Hz, 2H), 6.74 – 6.72 (m, 2H), 5.65 – 5.60 (m, 2H), 2.77 – 2.76 (m, 6H), 2.34 – 2.33 (m, 6H), 1.31 – 1.23 (m, 6H); $^{13}C\{^1H\}$ NMR (100 MHz, $CDCl_3$) δ 149.3, 149.2, 147.3, 141.6, 141.4, 141.2, 141.1, 140.9, 140.8, 140.3, 140.2, 139.0, 138.9, 138.2, 138.2, 137.9, 137.8, 137.7, 136.6, 134.0, 133.9, 133.9, 130.7, 130.7, 129.3, 129.2, 129.0, 127.3, 127.2, 127.2, 127.0, 126.8, 126.7, 126.7, 124.2, 124.2, 120.2, 120.1, 119.9, 119.6, 119.4, 118.3, 118.2, 115.9, 115.8, 50.0, 50.0, 22.0, 21.0, 19.1, 19.0, 18.9; HRMS (ESI) *m/z*: $[M + H]^+$ calcd for $C_{58}H_{45}$ 741.3521, found 741.3492 (error: -3.9 ppm).

12,12'-dimesityl-6,6'-biindeno[1,2-*b*]fluorene (5): DDQ (55 mg, 0.242 mmol, 2 equiv) was added to degassed solution of 1,2- dichloroethane (4 ml) of **10** (90 mg, 0.121 mmol) under N_2, and the reaction mixture was warmed to 80 °C using an oil bath for 2 h in a sealed tube. Once the starting material was consumed (monitored by TLC), the 1,2 dichloroethane was removed in vacuo and the crude was purified by silica gel column chromatography (EtOAc/hexane 2:100) to afford title product **5** as blue solid. (67 mg, 75% yield): R_f = 0.46 (5% EtOAc/hexanes); mp = 365-366°C; ^{1}H NMR (400 MHz, $CDCl_3$) δ 7.38 (d, *J* = 1.6 Hz, 1H), 7.36 (s, 3H), 7.32 – 7.28 (m, 3H), 7.27 (d, *J* = 1.3 Hz, 1H), 7.07 (d, *J* = 6.9 Hz, 2H), 7.04 (t, *J* = 1.8 Hz, 5H), 7.03 – 6.98 (m, 3H), 6.96 (dd, *J* = 3.6, 1.4 Hz, 1H), 6.94 – 6.92 (m, 3H), 6.70

(d, J = 7.2 Hz, 2H), 2.41 (s, 6H), 2.23 – 2.20 (m, 12H); $^{13}C\{^1H\}$ NMR (100 MHz, $CDCl_3$) δ 147.5, 144.0, 143.2, 139.4, 139.3, 138.4, 138.2, 138.2, 137.8, 137.2, 137.1, 136.2, 135.2, 129.9, 128.4, 128.0, 127.8, 127.7, 127.5, 123.7, 122.5, 120.8, 120.4, 119.8, 119.7, 21.3, 20.5, 20.5; HRMS (ESI) *m/z*: $[M]^+$ calcd for $C_{58}H_{42}$ 738.3287, found 738.3287 (error: 0 ppm).

1.3 NMR spectra

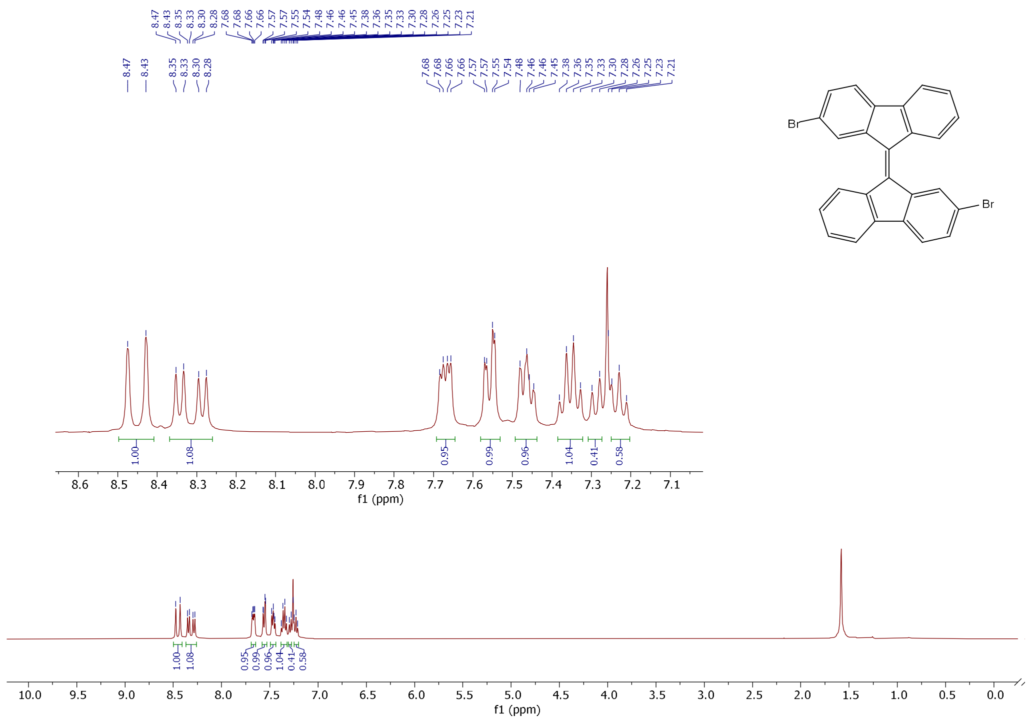

Fig. S1 ^1H NMR spectrum of **6** (in $CDCl_3$, 400 MHz, 298 K).

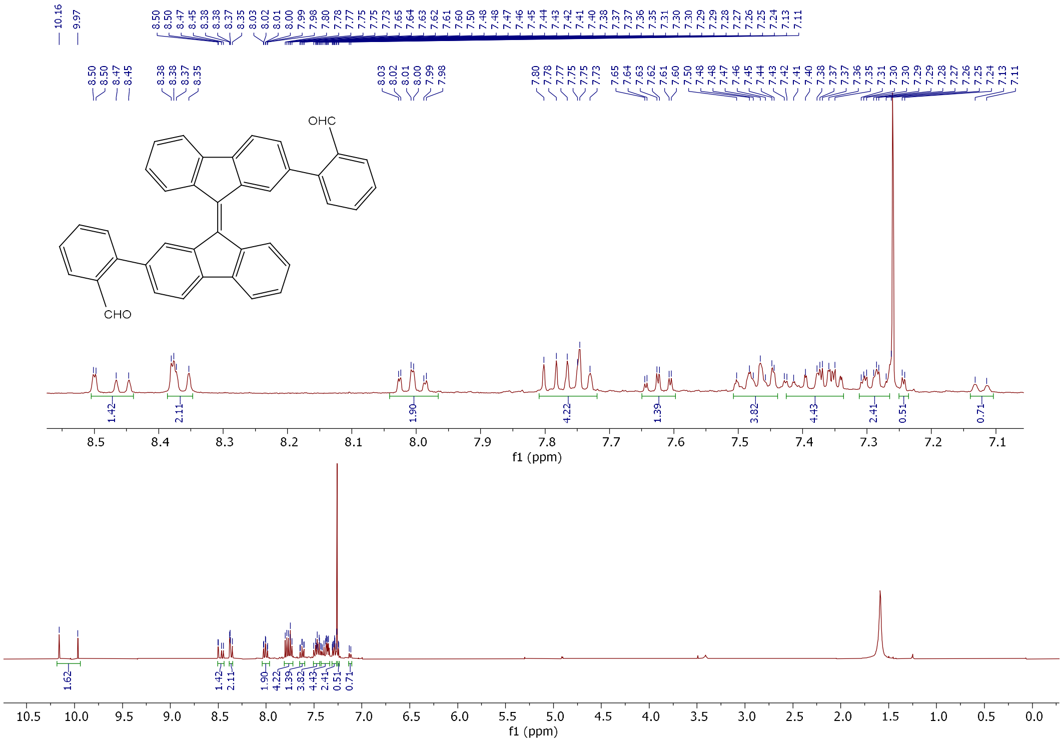

Fig. S2 ^{1}H NMR spectrum of **8** (in $CDCl_3$, 400 MHz, 298 K).

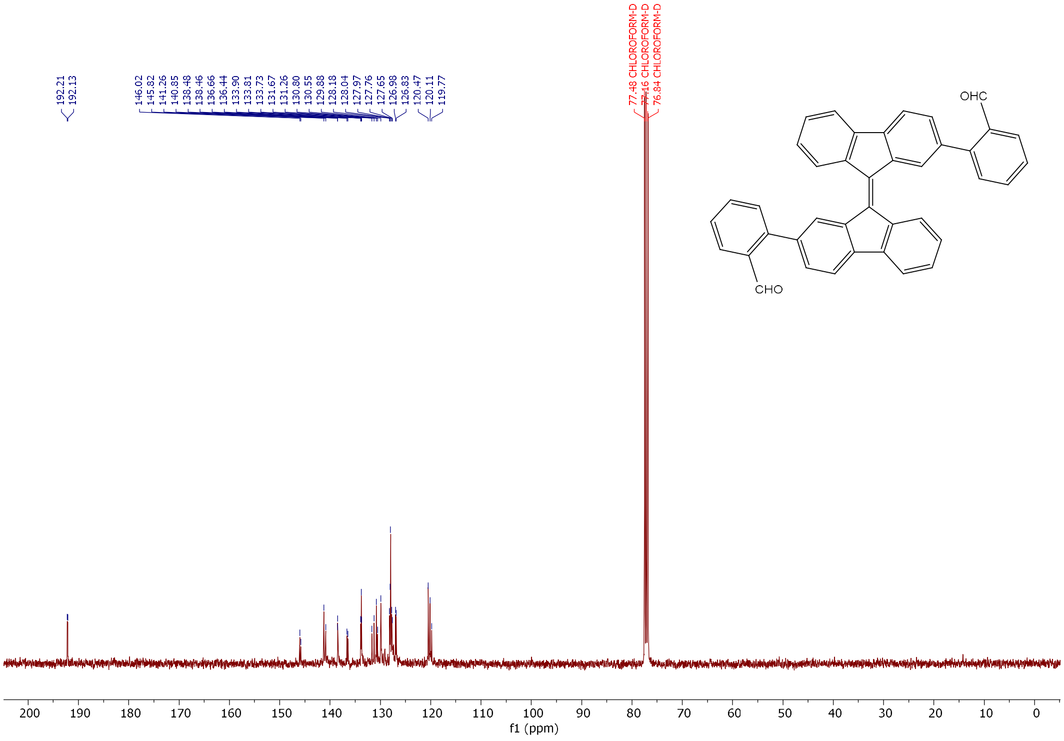

Fig. S3 $^{13}C\{^{1}H\}$ NMR spectrum of **8** (in $CDCl_3$, 100 MHz, 298 K).

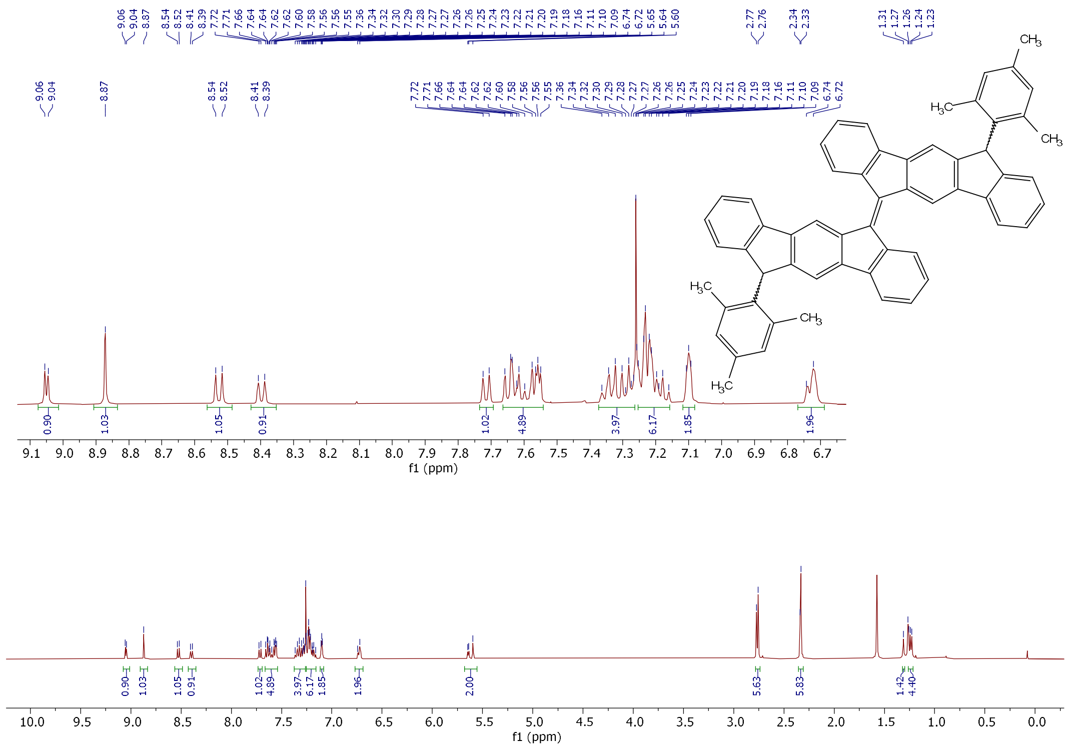

Fig. S4 ^{1}H NMR spectrum of **10** (in $CDCl_3$, 400 MHz, 298 K).

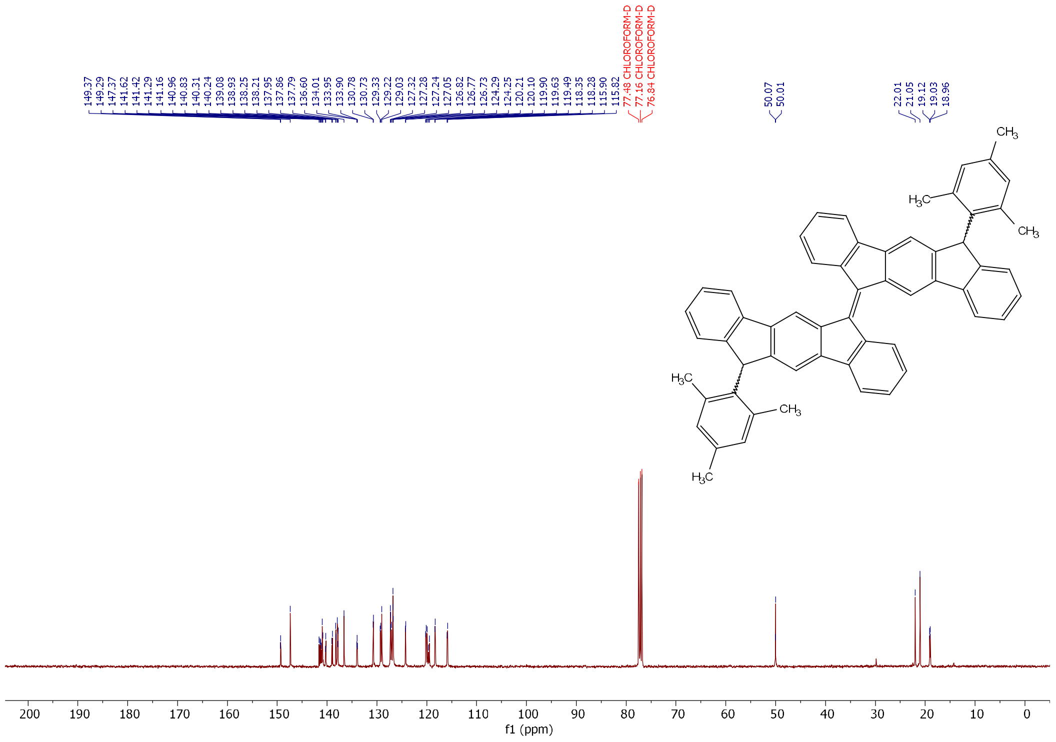

Fig. S5 $^{13}C\{^{1}H\}$ NMR spectrum of **10** (in $CDCl_3$, 100 MHz, 298 K).

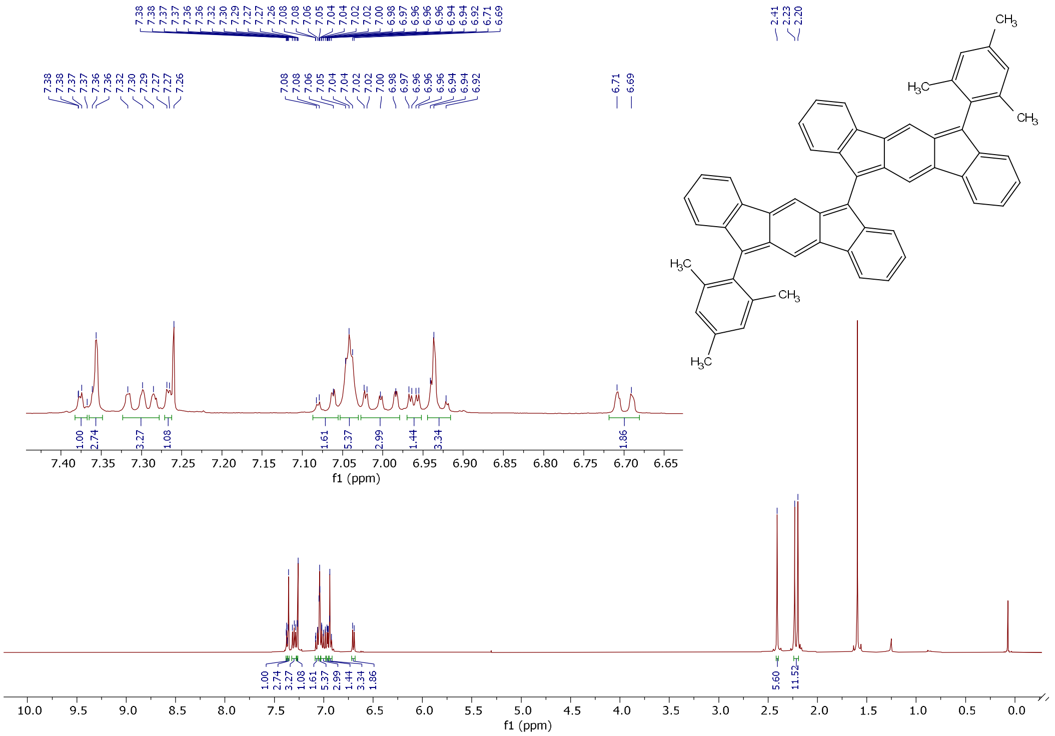

Fig. S6 ^{1}H NMR spectrum of **5** (in $CDCl_3$, 400 MHz, 298 K).

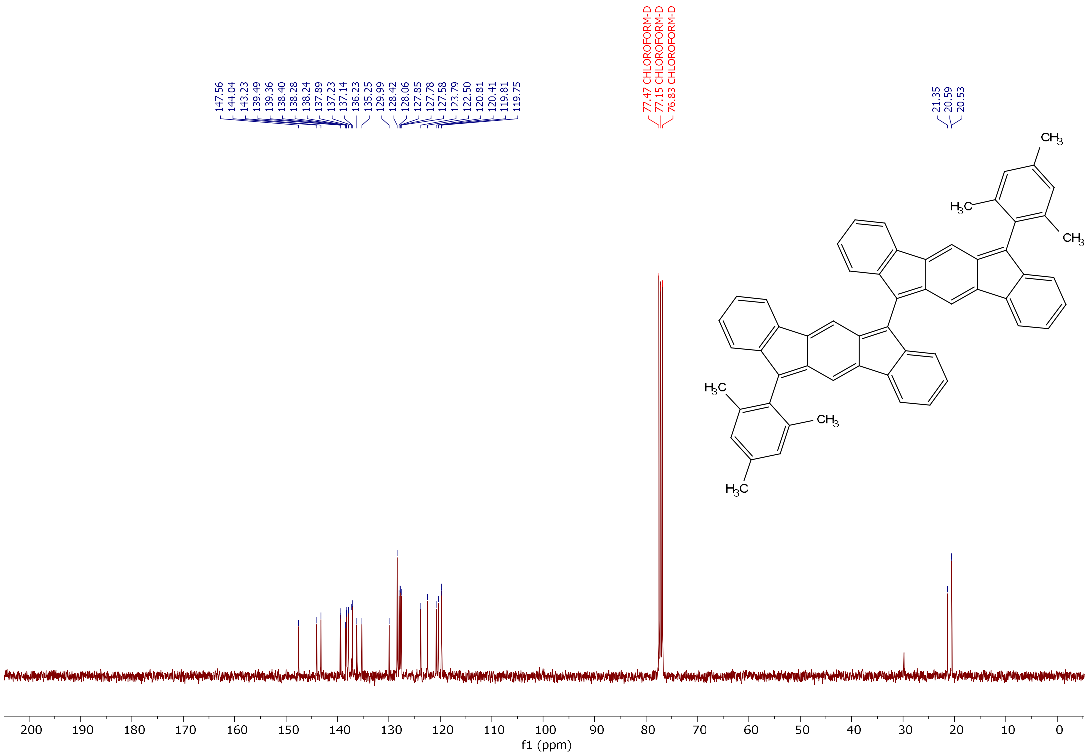

Fig. S7 $^{13}C\{^{1}H\}$ NMR spectrum of **5** (in $CDCl_3$, 100 MHz, 298 K).

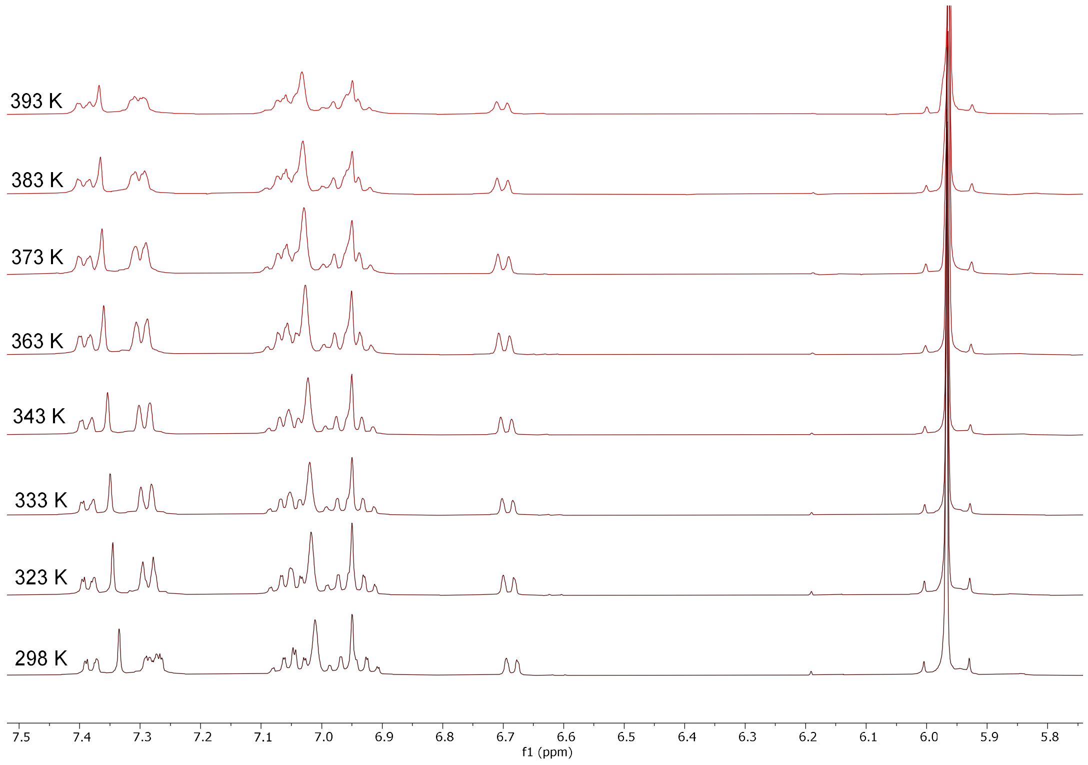

Fig. S8 Variable temperature ^{1}H NMR spectrum of **5** (in $C_2D_2Cl_4$, 400 MHz).

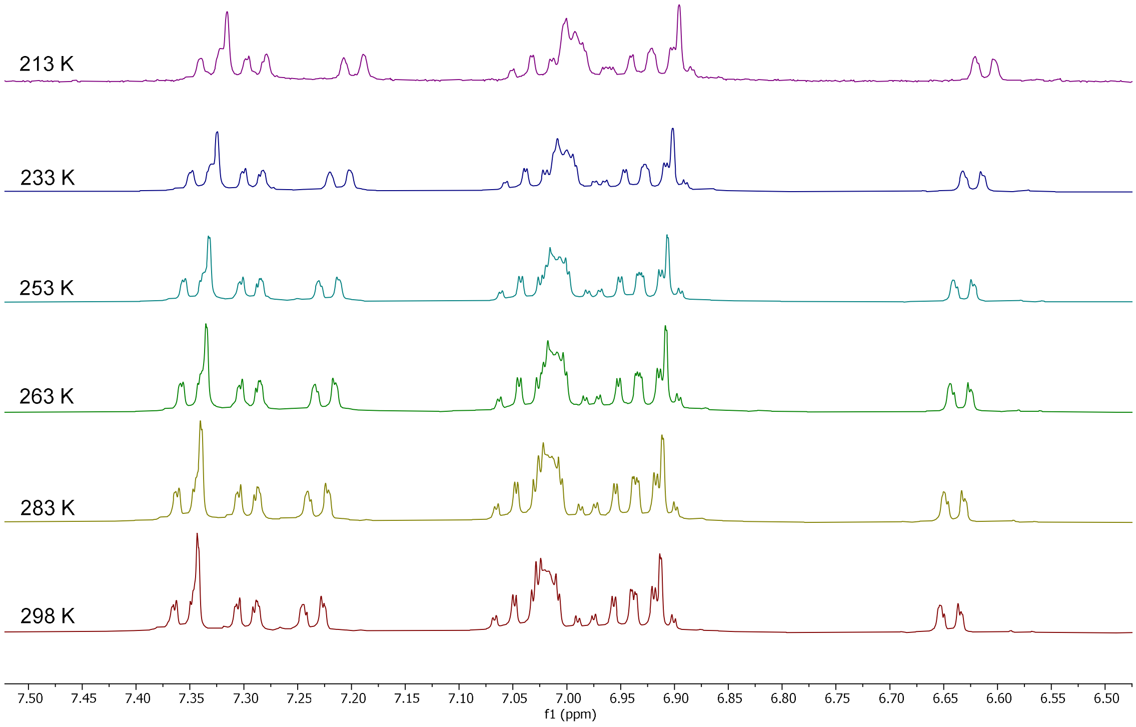

Fig. S9 Variable temperature ^{1}H NMR spectrum of **5** (in CD_2Cl_2, 400 MHz).

2. DFT calculations

Gas-phase density functional theory (DFT) calculations were performed with Gaussian 09 package using a high-performance computing cluster facility of IIT Ropar at the CAM-B3LYP level of theory with basis set 6-31G(d,p).[1] Optimization of the molecular geometries for **5**, was done by restricted CAM-B3LYP, and unrestricted CAM-B3LYP wave-functions using broken symmetry formalism.[2] The calculated NOON (natural orbital occupation number) value was based on the broken symmetry CAM-B3LYP method for ground state structure.[3] NICS (standard GIAO method)[4] values were calculated for the optimized structure at (U)BHandHLYP/6-31G(d,p) level, and the reported $NICS(1)_{zz}$ values are the average of two positions (1Å above and below the plane). Molecular orbital contributions for TDDFT calculation were determined using the GaussSum 3.0 package.[5]

Table S1. Relative energies for the optimized structure **5**

State	Hartree	kcal/mol
Singlet Closed-Shell CAM-B3LYP/6-31G(d,p)	-2234.270543	-1402024.874
Singlet Open-Shell UCAM-B3LYP /6-31G(d,p)	-2234.274569	-1402027.401
Triplet biradical UCAM-B3LYP /6-31G(d,p)	-2234.266828	-1402022.543

Compound 5 : $\Delta E_{Singlet(OS)-Triplet}$ = –4.85 kcal/mol; ΔE_{OS-CS} = –2.52 kcal/mol; ΔE_{CS-T} = –2.33 kcal/mol. Occupation numbers of HOMO-1 = 1.889, HOMO = 1.435, LUMO = 0.564, LUMO+1 = 0.111, affording a 26.8% diradical character (singlet diradical character index (y_0) = 0.268) and 0.7% tetraradical character (singlet tetraradical character index (y_1) = 0.007).

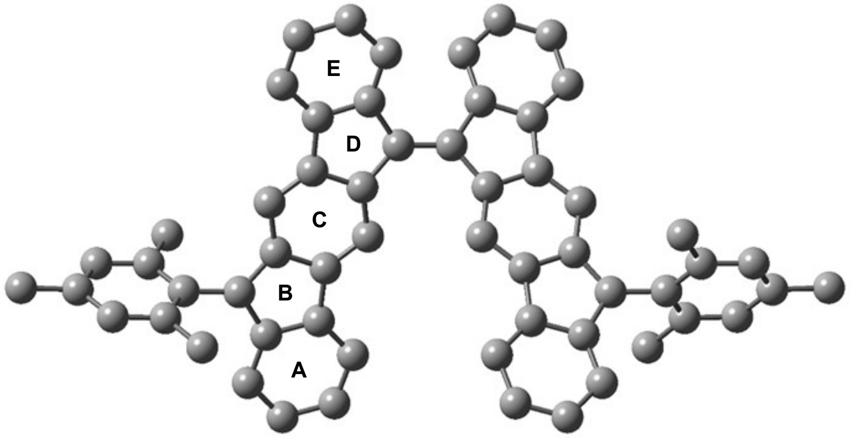

Fig. S10 Optimized structure of **5** in the open-shell ground state.

Table S2. NICS(1)$_{zz}$ values of each ring for **5**

**Rings**	**Closed shell 5**	**Open shell singlet 5**	**Open shell triplet 5**	**Closed shell 1**[6]
**A**	-18.18	-18.04	-16.63	-19.5
**B**	6.35	5.40	5.40	13.6
**C**	7.31	-2.0	-9.27	11.3
**D**	6.07	5.55	5.30	-
**E**	-18.34	-18.90	-19.48	-

TDDFT computation of compound 5

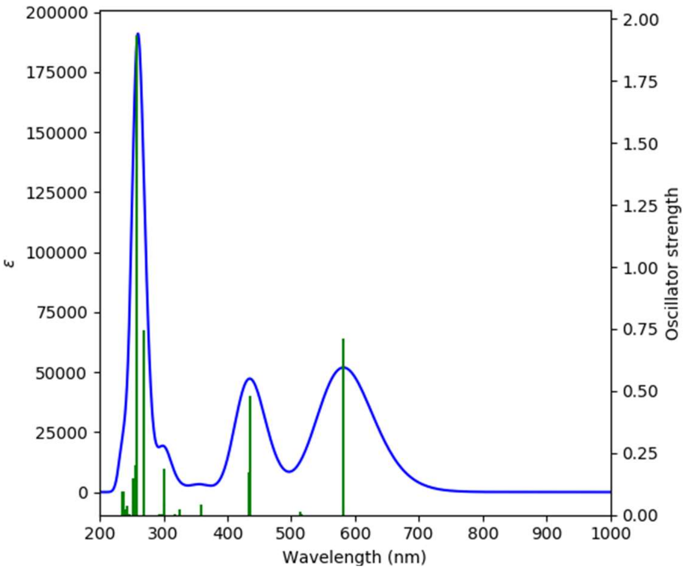

Fig. S11 Theoretical absorption of **5** at CAM-B3LYP/6-31G(d,p) level of theory.

Table S3. Summary of TDDFT calculation for **5**

Wavelength (nm)	Osc. Strength (*f*)	Major contributions
582.1	0.7125	HOMO->LUMO (95%)
516.5	0.0027	H-3->LUMO (55%), H-2->L+1 (23%), H-1->LUMO (19%)
513.7	0.015	H-3->L+1 (17%), H-2->LUMO (74%)
435.5	0.4826	HOMO->L+1 (87%)
433.4	0.1697	H-3->LUMO (27%), H-1->LUMO (64%)
358.0	0.043	H-3->L+1 (27%), H-1->L+1 (63%)
330.0	0.0003	H-3->L+1 (52%), H-2->LUMO (23%), H-1->L+1 (22%)
329.4	0.0005	H-3->LUMO (15%), H-2->L+1 (70%)
324.9	0.0001	H-5->L+1 (19%), H-4->LUMO (73%)
324.7	0.0214	H-5->LUMO (75%), H-4->L+1 (20%)
323.9	0.0009	H-1->L+3 (12%), HOMO->L+2 (66%)
317.5	0.0019	H-1->L+2 (18%), HOMO->L+3 (60%)
300.4	0.1872	H-10->LUMO (61%), H-9->L+1 (16%)
300.3	0.0058	H-8->LUMO (50%), H-7->LUMO (22%)
300.1	0.0507	H-10->L+1 (10%), H-9->LUMO (72%)
297.7	0.0036	H-11->LUMO (17%), H-7->L+1 (12%), H-6->LUMO (56%)
295.7	0.0007	H-8->LUMO (19%), H-7->LUMO (57%), H-6->L+1 (13%)
293.4	0.0049	H-11->LUMO (39%), H-10->LUMO (12%), H-8->L+1 (12%), H-6->LUMO (18%)
269.5	0.7444	H-12->LUMO (17%), H-3->L+2 (29%), H-2->L+3 (27%)
258.8	1.9368	H-3->L+3 (18%), H-2->L+2 (41%), H-1->L+3 (13%)
256.3	0.2009	H-12->LUMO (55%), H-2->L+3 (10%)
252.7	0.1483	H-13->LUMO (51%)
247.3	0.0048	H-11->LUMO (11%), H-8->L+1 (26%), H-1->L+2 (19%), HOMO->L+3 (12%)
244.3	0	H-11->L+1 (17%), H-1->L+3 (14%)
242.6	0.0349	H-9->L+1 (10%), H-8->L+1 (28%), H-1->L+2 (15%)
239.6	0.0242	H-14->LUMO (46%), H-9->L+1 (16%)
238.5	0.0071	H-15->LUMO (33%), HOMO->L+6 (24%)
237.8	0.0948	H-14->LUMO (11%), H-9->L+1 (32%)
236.9	0.0393	H-11->L+1 (30%), H-10->L+1 (16%)
236.3	0.0972	H-10->L+1 (32%), H-5->L+1 (14%)

3. X-ray crystallographic analyses

Single crystals of **5** were selected using paratone oil and mounted on glass fiber with the help of gum. The intensity data and geometric parameters of these crystals were garnered with the help of Bruker D8 Venture X-ray diffractometer having a micro-focus sealed X-ray tube Mo-

Kα (λ = 0.71073 Å) source of X-rays along with a PHOTON 100 detector with inclining Phi and Omega (width of 0.5 for one frame) working at a scan speed of 10 s per frame. The crystal was kept at 298 K during data collection. Data acquisition as well as extraction of data was accomplished by utilizing Bruker Apex-3 and Bruker SAINT software packages using a narrow -frame algorithm.[7] By utilizing OLex2,[8] the crystal structure was solved with the help of olex2.solve[9] structure solution program by employing intrinsic Phasing methods and crystal structure refinement was done with the SHELXL[10] refinement package by putting into use Least Squares minimization. Refinement of all non-hydrogen atoms was completed with the help of anisotropic thermal parameters.

Table S4. X-ray crystallographic information of **5**

CCDC No.	2362686
Empirical formula	$C_{58}H_{42}$
Formula weight	738.91
Temperature/K	298.00
Crystal system	Monoclinic
Space group	$P2_1$
a/Å	14.265(3)
b/Å	8.009(2)
c/Å	19.200(4)
α/°	90
β/°	106.474(6)
γ/°	90
Volume/Å^3	2103.5(8)
Z	2
ρ_{calc}g/cm^3	1.167
μ/mm^{-1}	0.066
F(000)	780.0
Crystal size/mm^3	0.315 × 0.045 × 0.023
Radiation	MoKα (λ = 0.71073)
2Θ range for data collection/°	4.424 to 51.362
Index ranges	$-17 \le h \le 17$, $-9 \le k \le 9$, $-23 \le l \le 23$
Reflections collected	35050
Independent reflections	7972 [R_{int} = 0.0901, R_{sigma} = 0.0841]
Data/restraints/parameters	7972/1/529
Goodness-of-fit on F^2	1.023
Final R indexes [I>=2σ (I)]	R_1 = 0.0657, wR_2 = 0.1376

Final R indexes [all data]	$R_1 = 0.1116$, $wR_2 = 0.1557$
Largest diff. peak/hole / e Å^{-3}	0.35/-0.16
Flack parameter	5.3(10)

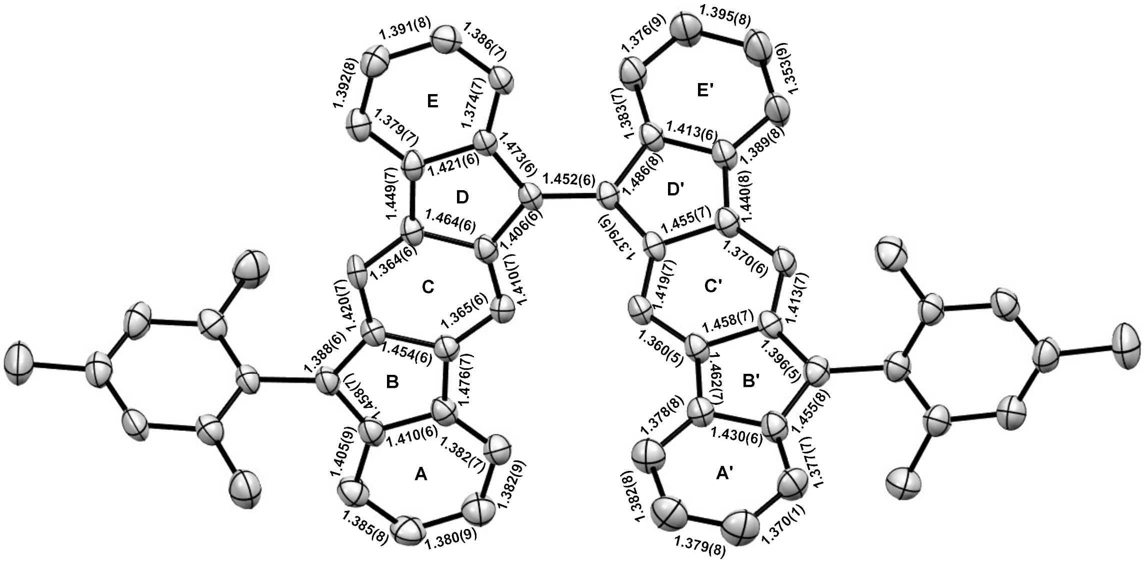

Fig. S12 Full crystallographic bond length analyses of **5** with e.s.d values.

4. Photostability test

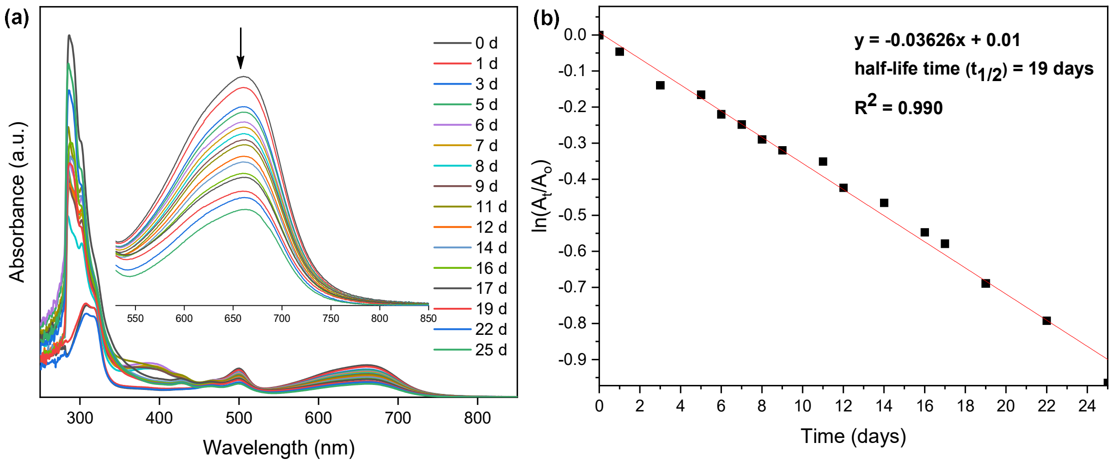

Fig. S13 a) Absorption spectral changes under ambient light conditions for compound **5** in toluene (0 days to 25 days); b) Fitting with first-order kinetics.

The half-life ($t_{1/2}$) of was determined according to the following equation:

$$t_{1/2} = \frac{ln2}{0.03626} = 19 \text{ days}$$

To check the photostability of final compound **5**, solution of known concentration of **5** in toluene was exposed to ambient light (under normal lab light condition and temperature). It leads to the gradual decomposition of **5** in solution, resulting in the decrease of the absorbance at longer wavelength (661 nm) region.

5. EPR measurement and Bleaney-Bowers plot

To get the ground state spin multiplicity and the energy separation between the singlet and triplet state, VT -EPR experiments were carried out using the solid sample of **5** displaying g_e = 2.0043. The modified Bleaney-Bowers equation was used to fit the *IT* against the *T* data points to get the energy separation between the singlet and triplet spin state of the diradicaloid species.

$$IT = \frac{2\rho N_A g^2 \beta^2}{k_b} * \frac{1}{3 + \exp\left(-\frac{2J}{k_b T}\right)} + \frac{(1-\rho) N_A g^2 \beta^2}{2k_b}$$

Here, *2J* is the energy separation between singlet and triplet state ($\Delta E_{S\text{-}T}$), '*I* 'is the EPR intensity, 'T' is the temperature in Kelvin scale, *g* is the g factor, N_A is the Avogadro constant, β is the Bohr magneton, k_b is the Boltzmann constant, and ρ is the paramagnetic purity.

The *IT* vs. *T* data set was fitted according to the below equation using Origin 2021 software package.

$$IT = \frac{a}{3 + \exp\left(-\frac{b}{T}\right)} + c$$

$$a = \frac{2\rho N_A g^2 \beta^2}{k_b}\ ;\ b = \frac{2J}{k_b}\ ;\ c = \frac{(1-\rho) N_A g^2 \beta^2}{2k_b}$$

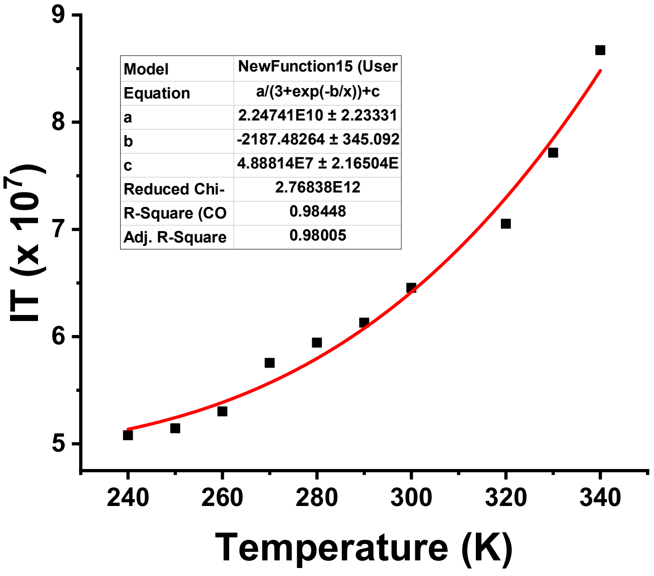

Fig. S14 Bleaney-Bowers plot for **5**.

b = −2187.48 ± 345.09;

$2J = k_B * b$ = −4.35 ± 0.6 kcal/mol

The experimental energy gap ($\Delta E_{S\text{-}T}$) between the singlet ground state and the excited triplet state is −4.35 ± 0.6 kcal/mol.

6. Appendix

ACID plot of **5**

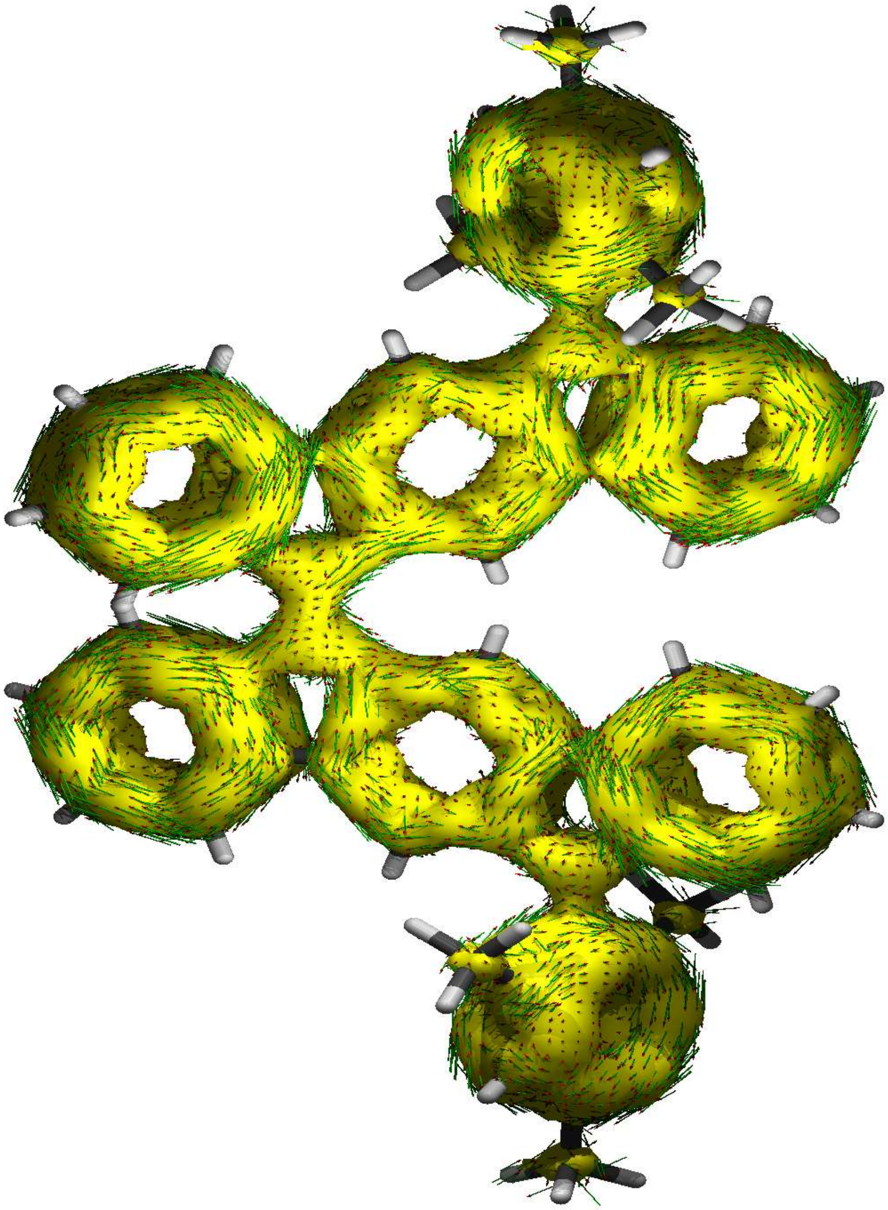

The ACID plots of **5** at isosurface values of 0.0353 (left image) and 0.055 (right image) are shown below. The critical isosurface value (CIV) for central benzenoid ring C is found to be 0.0353. The CIV for outer benzene rings A as well as E is 0.055.

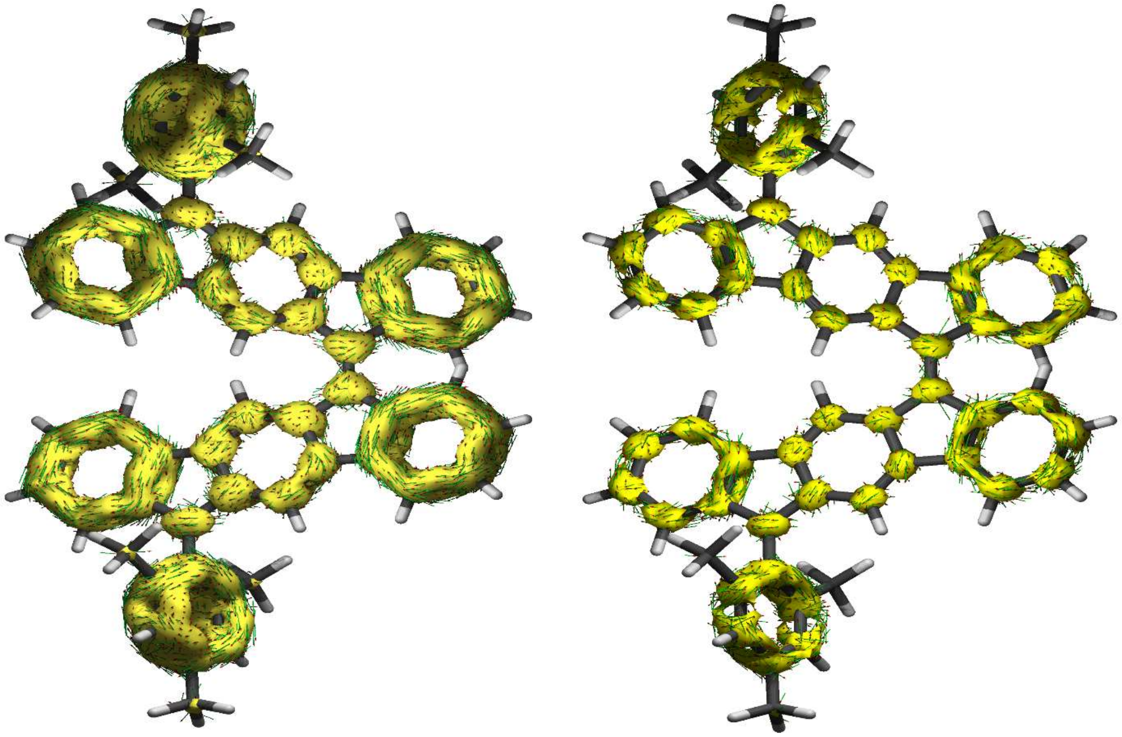

Cartesian coordinates (in Angstroms) for the optimized structure:

Singlet closed-shell structure of 5

Atom type	x	y	z
C	0.69977800	2.39780700	-0.19530100
C	3.88572500	-0.23586400	0.28018500
C	-1.62457500	1.40593100	-0.03858200
C	-1.37472600	3.48405300	0.92985500
C	2.72218600	3.11881700	-1.13529100
C	-0.69979600	2.39779700	0.19543100
C	0.88612700	4.67680300	-1.44827200
H	-0.15294000	4.95642900	-1.31419500
C	1.62459200	1.40598700	0.03876600
C	1.37465800	3.48402200	-0.92983600
C	2.59675000	-0.62562700	0.85940700
C	6.25060000	-1.19153200	0.15628800
C	2.81033600	-1.92540800	1.50522300
C	2.91842100	1.80401500	-0.52043900
C	-4.01169000	1.01706600	0.41470000
H	-4.97300600	1.30532800	0.83041800
C	-0.88623500	4.67687500	1.44823000
H	0.15283400	4.95650700	1.31417700
C	4.16571400	-2.27043200	1.30580100

C	4.81970600	-1.20021800	0.54167500
C	-2.91844400	1.80399100	0.52051200
C	-1.50534400	0.16307300	-0.75621400
H	-0.55427900	-0.11336500	-1.19978100
C	1.50541800	0.16318700	0.75650800
H	0.55438500	-0.11322000	1.20016300
C	4.01167200	1.01709000	-0.41466800
H	4.97294900	1.30530500	-0.83050500
C	3.58184100	3.94997300	-1.83368400
H	4.61844200	3.67030200	-1.99532300
C	-2.72226100	3.11884400	1.13526900
C	-2.59666500	-0.62575200	-0.85913100
C	-3.88568900	-0.23594200	-0.28004500
C	7.17693900	-0.45174600	0.90902000
C	1.96700000	-2.76950400	2.20570300
H	0.92361300	-2.51353100	2.36275700
C	-4.81965500	-1.20029600	-0.54157300
C	-2.81020000	-1.92558500	-1.50486400
C	3.09072800	5.15462800	-2.33464700
H	3.75175300	5.81779900	-2.88296500
C	1.75730500	5.51061700	-2.14627200
H	1.38995400	6.44737000	-2.55256400
C	-7.17691400	-0.45225800	-0.90963800
C	2.48282600	-3.96389500	2.71206800
H	1.83364800	-4.63591400	3.26373500
C	3.81993000	-4.30250400	2.51933100
H	4.20002800	-5.23505200	2.92366200
C	-6.68287300	-1.93979500	0.95101200
C	-6.25060200	-1.19156600	-0.15637000
C	8.96661300	-1.19820500	-0.56121300
C	-4.16560000	-2.27057700	-1.30554500
C	6.68278900	-1.94028000	-0.95080500
C	4.67542300	-3.45675800	1.81449100
H	5.71902900	-3.71678000	1.66649200
C	6.73984500	0.34427000	2.11240300
H	7.60368000	0.73887800	2.65120800
H	6.15602400	-0.26807700	2.80518400
H	6.10646400	1.18969500	1.82810300
C	-3.81972300	-4.30276800	-2.51884300
H	-4.19978900	-5.23535400	-2.92311600
C	8.51857500	-0.46989000	0.53646800
H	9.23425300	0.09767900	1.12575300
C	-8.03297600	-1.92891500	1.28962000
H	-8.36352100	-2.50862200	2.14772000
C	-1.96680800	-2.76975100	-2.20519100
H	-0.92340200	-2.51380800	-2.36216800
C	-6.73967500	0.34317300	-2.11335200
H	-6.10594900	1.18846200	-1.82942200
H	-7.60342600	0.73789400	-2.65221000
H	-6.15615300	-0.26964200	-2.80597500
C	-4.67527400	-3.45695100	-1.81415600
H	-5.71889100	-3.71695700	-1.66621000
C	8.03282000	-1.92942100	-1.28962600
H	8.36329500	-2.50951300	-2.14749400
C	-2.48260100	-3.96418500	-2.71149600
H	-1.83337900	-4.63625800	-3.26304500

C	-1.75745600	5.51072700	2.14613300
H	-1.39013600	6.44751200	2.55237900
C	-8.51858800	-0.47035000	-0.53728700
H	-9.23423700	0.09685500	-1.12696100
C	-8.96672000	-1.19816800	0.56070800
C	-3.58195800	3.95003700	1.83356500
H	-4.61856400	3.67036700	1.99517600
C	5.70238100	-2.73544900	-1.77385600
H	4.93047200	-2.09003700	-2.20318000
H	5.18565700	-3.48539800	-1.16826400
H	6.20803600	-3.25060400	-2.59300300
C	-3.09088300	5.15473400	2.33447000
H	-3.75194300	5.81793200	2.88271300
C	-5.70251900	-2.73450400	1.77457000
H	-5.18560100	-3.48463900	1.16936900
H	-6.20825900	-3.24939000	2.59383400
H	-4.93076100	-2.08883200	2.20377400
C	-10.41777300	-1.17700600	0.96295400
H	-10.71112700	-2.11100400	1.44831900
H	-11.06893200	-1.02417300	0.09887700
H	-10.61862800	-0.36499000	1.67022200
C	10.41758600	-1.17714900	-0.96375200
H	10.61769500	-0.36717500	-1.67356900
H	10.71158200	-2.11240600	-1.44631000
H	11.06876100	-1.02117700	-0.10025700

Singlet open-shell structure of 5

Atom type	**x**	**y**	**z**
C	0.68690400	2.48431100	-0.16817900
C	3.85768100	-0.24256500	0.24878000
C	-1.61807300	1.41430900	-0.00657500
C	-1.43363800	3.59971900	0.78087200
C	2.77154800	3.19540300	-0.97563000
C	-0.68684600	2.48431800	0.16828100
C	1.00800600	4.83001900	-1.27413900
H	-0.02699100	5.13712700	-1.18402300
C	1.61810800	1.41427500	0.00663300
C	1.43371900	3.59971900	-0.78072800
C	2.56992500	-0.65113100	0.75239200
C	6.23824900	-1.22944000	0.15949700
C	2.74358800	-1.98711100	1.33357700
C	2.91082400	1.83330200	-0.45819800
C	-4.01538900	1.02776000	0.35338800
H	-4.99175700	1.34807800	0.70503100
C	-1.00789600	4.82998600	1.27433700
H	0.02711100	5.13706800	1.18424600
C	4.10453000	-2.34383900	1.16671200
C	4.79710600	-1.25591500	0.49560200
C	-2.91078300	1.83335200	0.45825700

C	-1.47283600	0.16152300	-0.65241600
H	-0.51554000	-0.13790300	-1.06380500
C	1.47285000	0.16148200	0.65246000
H	0.51555300	-0.13792000	1.06386700
C	4.01541200	1.02768300	-0.35335700
H	4.99179200	1.34799000	-0.70497900
C	3.68488700	4.02795700	-1.60176500
H	4.71247000	3.71152000	-1.75200700
C	-2.77147900	3.19542900	0.97574400
C	-2.56993400	-0.65105800	-0.75238600
C	-3.85768900	-0.24246700	-0.24879900
C	7.13783600	-0.53003800	0.98066000
C	1.86273400	-2.85541000	1.95203400
H	0.81718100	-2.59313000	2.08223900
C	-4.79712900	-1.25581100	-0.49562500
C	-2.74360500	-1.98704200	-1.33355500
C	3.25864400	5.27493600	-2.05230300
H	3.96201400	5.93979500	-2.54291500
C	1.93039900	5.66569600	-1.89725900
H	1.60660100	6.63005700	-2.27480100
C	-7.13777200	-0.52935300	-0.98032800
C	2.34134200	-4.08515500	2.41044400
H	1.66057200	-4.77550100	2.89768000
C	3.68085800	-4.43643200	2.25166500
H	4.02980700	-5.39638600	2.61799700
C	-6.70858900	-1.91493200	0.97272900
C	-6.23827100	-1.22934600	-0.15954100
C	8.97623900	-1.19189000	-0.46942500
C	-4.10455200	-2.34375500	-1.16670200
C	6.70844900	-1.91438000	-0.97322400
C	4.57513500	-3.56882500	1.62970000
H	5.61994800	-3.83759700	1.50804900
C	6.65812900	0.19459900	2.21237900
H	7.50035300	0.58898600	2.78448300
H	6.08329200	-0.46827700	2.86534400
H	6.00242600	1.03213200	1.95725100
C	-3.68088900	-4.43633800	-2.25168500
H	-4.02984800	-5.39628000	-2.61804000
C	8.49076000	-0.52539300	0.65134600
H	9.18509100	0.01041500	1.29349700
C	-8.06883900	-1.88382100	1.26620500
H	-8.42854900	-2.41522500	2.14366100
C	-1.86275200	-2.85534500	-1.95201000
H	-0.81719700	-2.59306800	-2.08221000
C	-6.65797400	0.19595200	-2.21162200
H	-6.00218300	1.03327100	-1.95602100
H	-7.50015600	0.59075800	-2.78349700
H	-6.08320800	-0.46660700	-2.86496900
C	-4.57516600	-3.56872400	-1.62973200
H	-5.61999200	-3.83747400	-1.50813800
C	8.06867100	-1.88319900	-1.26675600
H	8.42829900	-2.41413300	-2.14453300
C	-2.34136300	-4.08508200	-2.41043200
H	-1.66059500	-4.77543300	-2.89766200
C	-1.93027400	5.66566400	1.89748000
H	-1.60645500	6.63000200	2.27506200

C	-8.49069900	-0.52480500	-0.65108600
H	-9.18495700	0.01144600	-1.29295000
C	-8.97630100	-1.19196000	0.46925800
C	-3.68480200	4.02798400	1.60190100
H	-4.71239500	3.71156800	1.75212200
C	5.75780300	-2.66391700	-1.87093600
H	4.98302100	-2.00170400	-2.26844500
H	5.24305200	-3.46465500	-1.33224200
H	6.28813600	-3.10980200	-2.71473800
C	-3.25853100	5.27493400	2.05249200
H	-3.96189000	5.93979100	2.54312300
C	-5.75809800	-2.66519900	1.86999700
H	-5.24506100	-3.46706100	1.33131500
H	-6.28822000	-3.10978100	2.71461900
H	-4.98194900	-2.00385700	2.26625200
C	-10.43917500	-1.14678500	0.82388700
H	-10.75595400	-2.06276500	1.32862300
H	-11.06123900	-1.01620100	-0.06484300
H	-10.65504800	-0.31161700	1.49904600
C	10.43909300	-1.14671300	-0.82414000
H	10.65457900	-0.31276800	-1.50093100
H	10.75629000	-2.06351800	-1.32712500
H	11.06109800	-1.01415300	0.06433100

Triplet structure of 5

Atom type	**x**	**y**	**z**
C	0.66879300	2.53727100	-0.14738400
C	3.85068300	-0.24251800	0.23667500
C	-1.61705300	1.41167500	0.01585400
C	-1.47169300	3.66742900	0.67272800
C	2.79941500	3.23507200	-0.86234500
C	-0.66883900	2.53722500	0.14757300
C	1.09133600	4.91773000	-1.15594400
H	0.06112600	5.24552700	-1.10279100
C	1.61704900	1.41173900	-0.01569000
C	1.47159300	3.66747700	-0.67261300
C	2.56702300	-0.66544900	0.69029100
C	6.24457400	-1.25170900	0.16074300
C	2.72076900	-2.02220300	1.22686000
C	2.90507300	1.84666700	-0.41469500
C	-4.02173600	1.03374100	0.30790400
H	-5.00482700	1.37645300	0.61650100
C	-1.09146900	4.91768300	1.15607200
H	-0.06124900	5.24546500	1.10301400
C	4.08702700	-2.38460500	1.08084600
C	4.80013400	-1.29127500	0.47247200
C	-2.90511900	1.84659900	0.41473400
C	-1.46257300	0.15707300	-0.58928800
H	-0.50398000	-0.16021600	-0.98076900
C	1.46262300	0.15718500	0.58955700
H	0.50405800	-0.16007100	0.98113700
C	4.02170500	1.03382700	-0.30789900

H	5.00477000	1.37651300	-0.61660200
C	3.75099300	4.06323000	-1.43905800
H	4.77090100	3.71938100	-1.58012700
C	-2.79952800	3.23501900	0.86235500
C	-2.56696000	-0.66557000	-0.69006800
C	-3.85066300	-0.24263300	-0.23658200
C	7.12507400	-0.54922800	1.00063400
C	1.81888800	-2.90501800	1.79075400
H	0.77202600	-2.63968100	1.90335500
C	-4.80008100	-1.29142100	-0.47239600
C	-2.72063900	-2.02236100	-1.22656200
C	3.37146500	5.33370600	-1.85823800
H	4.10362000	5.99513200	-2.30987400
C	2.04770100	5.74874500	-1.73040700
H	1.75327500	6.72796400	-2.09340400
C	-7.12494900	-0.54936600	-1.00092300
C	2.27592400	-4.15526300	2.21653600
H	1.57693100	-4.85655900	2.66039000
C	3.61780800	-4.51360900	2.08027100
H	3.94829300	-5.48926900	2.42125100
C	-6.73950200	-1.92084300	0.97120500
C	-6.24456000	-1.25182100	-0.16085100
C	8.99171600	-1.17907100	-0.42726300
C	-4.08690300	-2.38477400	-1.08064600
C	6.73935700	-1.92082900	-0.97135400
C	4.53417800	-3.63402400	1.51439900
H	5.57975100	-3.90752100	1.41132200
C	6.61971100	0.15456600	2.23409600
H	7.44726200	0.57354000	2.81010000
H	6.06598700	-0.53025400	2.88283400
H	5.93690000	0.97077300	1.98140400
C	-3.61756700	-4.51383600	-2.07989300
H	-3.94799900	-5.48952600	-2.42083600
C	8.48244800	-0.52735400	0.69174500
H	9.16130900	0.01004700	1.34895100
C	-8.10365900	-1.87259400	1.24365500
H	-8.48184500	-2.39065500	2.12132200
C	-1.81869500	-2.90519700	-1.79033100
H	-0.77183100	-2.63984500	-1.90286100
C	-6.61939500	0.15425300	-2.23440600
H	-5.93601200	0.96999300	-1.98177700
H	-7.44680000	0.57379000	-2.81020800
H	-6.06624000	-0.53086300	-2.88332800
C	-4.53399700	-3.63423200	-1.51415500
H	-5.57957200	-3.90774200	-1.41113200
C	8.10345000	-1.87262300	-1.24399100
H	8.48150800	-2.39072800	-2.12169300
C	-2.27567400	-4.15547100	-2.21607200
H	-1.57663800	-4.85678600	-2.65983000
C	-2.04788600	5.74871400	1.73043400
H	-1.75349100	6.72793700	2.09344300
C	-8.48233500	-0.52742200	-0.69221100
H	-9.16110000	0.00995300	-1.34954200
C	-8.99177900	-1.17906700	0.42679100
C	-3.75115500	4.06319000	1.43896400
H	-4.77107700	3.71935200	1.57995500

C	5.81109600	-2.67065100	-1.89224400
H	5.01699300	-2.02001000	-2.27018000
H	5.32043900	-3.50291500	-1.37955000
H	6.35474200	-3.07441800	-2.74871500
C	-3.37166000	5.33367700	1.85815000
H	-4.10385800	5.99511200	2.30970300
C	-5.81138000	-2.67051700	1.89235400
H	-5.31947000	-3.50192700	1.37949700
H	-6.35541600	-3.07540400	2.74804800
H	-5.01823400	-2.01939700	2.27150200
C	-10.45913600	-1.11482400	0.75918200
H	-10.79158500	-2.01939000	1.27428100
H	-11.06705300	-0.99344800	-0.14058900
H	-10.67703400	-0.26657600	1.41717900
C	10.45901200	-1.11501900	-0.75995400
H	10.67609400	-0.26979900	-1.42210400
H	10.79231800	-2.02175500	-1.27069700
H	11.06681600	-0.98874400	0.13920300

DFT-optimized C-C bond lengths of ground state open-shell structure **5**

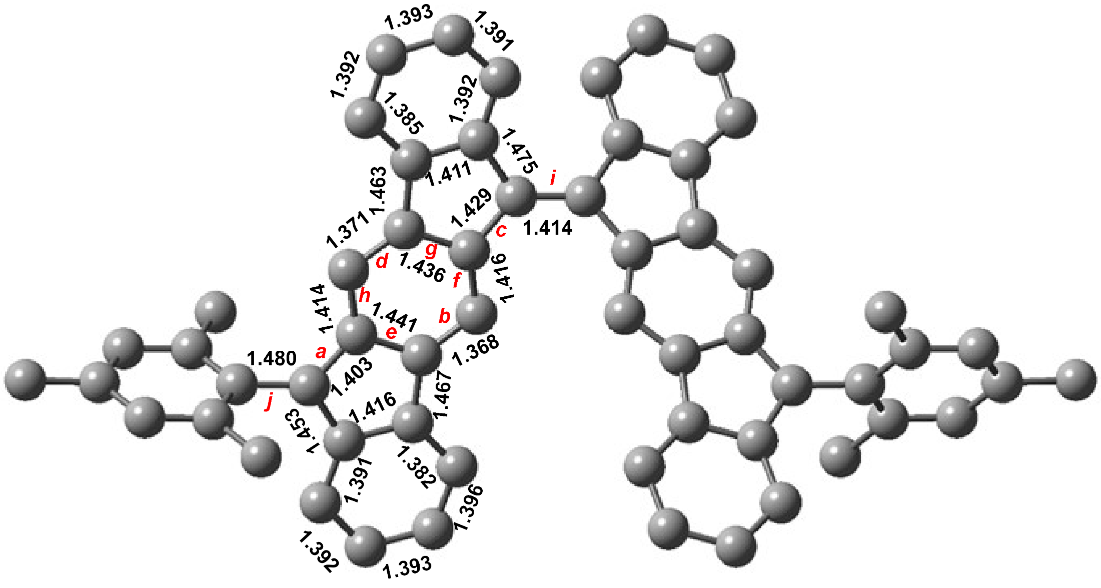

Comparison of X-ray crystallographic C-C bond lengths of **4** and **5**

Compound 4:

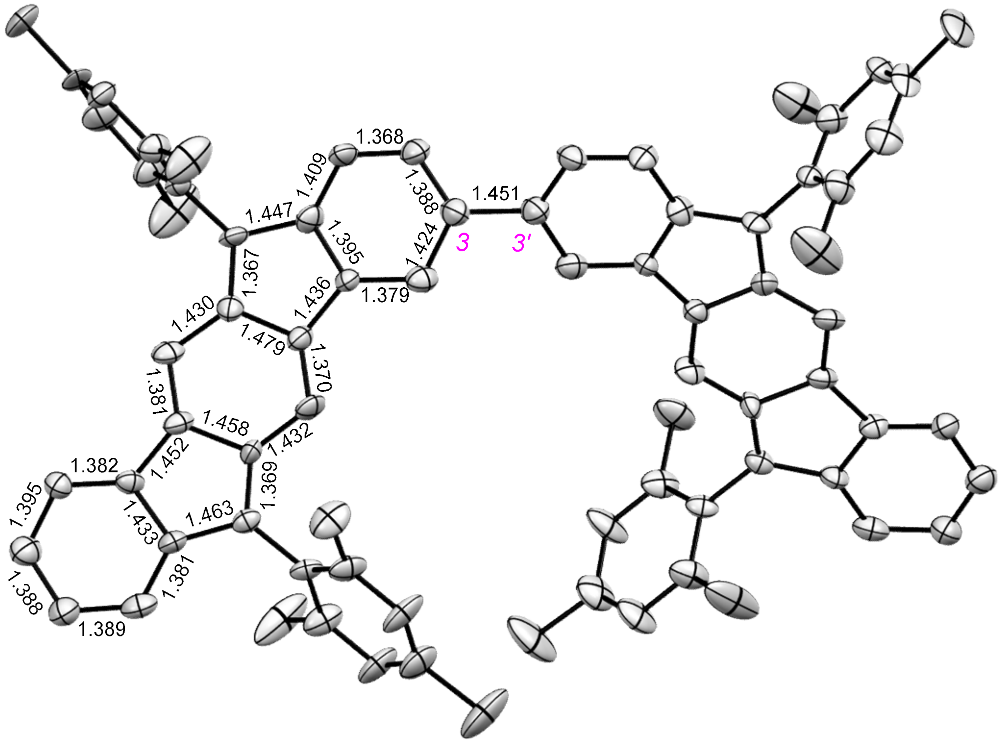

Compound 5:

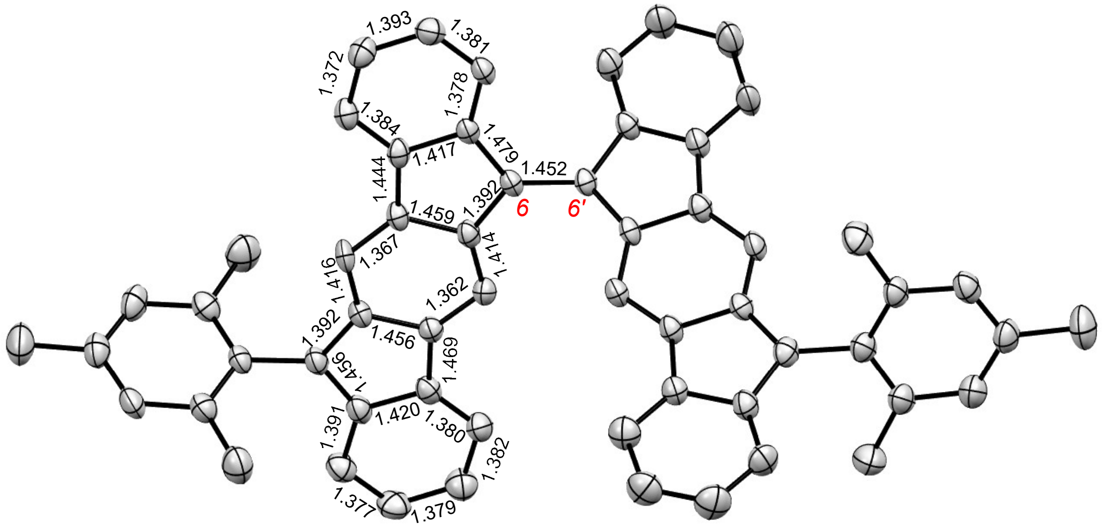

Explanation of four-stage redox amphotericity:

The diradicaloid ground state ($y_0 = 0.268$) of **5** is clearly evident from the cyclic voltammetry and differential pulse voltammetry (Fig. 5b). The excellent reversibility during oxidation (facile electron ejection) and reduction (facile electron injection) events showing sequential one-electron transfer processes can be explained through the diradical-like structure **5-OS1**, as shown below. During reduction, electrons can be injected easily at lower reduction potential (low-lying LUMO = –3.80 eV) as formation of locally aromatic cyclopentadienyl anions (shown below) in the five membered rings can be favored. During oxidation, the double bond arrangement in benzene rings became such that it avoids formation of locally antiaromatic cyclopentadienyl cation (shown below) in the five membered rings.

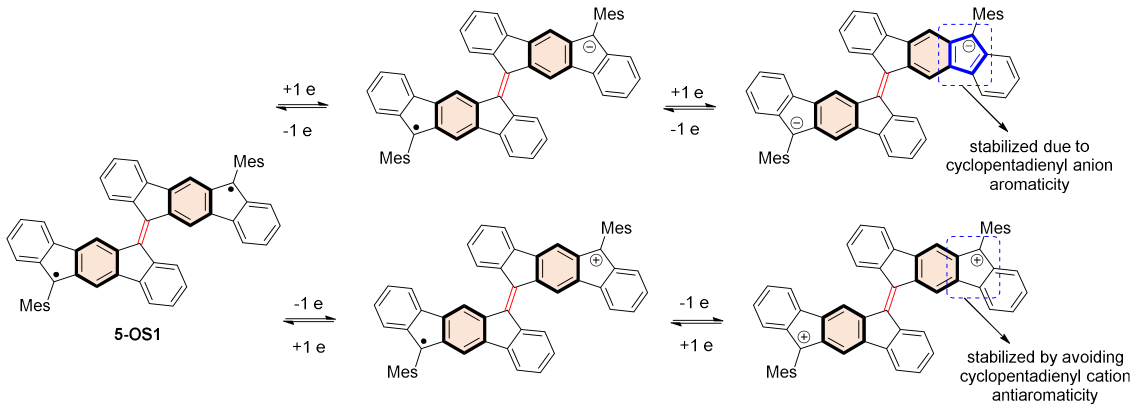

6. References

[1] Gaussian 09, *Revision B.01*, M. J. Frisch, G. W. Trucks, H. B. Schlegel, G. E. Scuseria, M. A. Robb, J. R. Cheeseman, G. Scalmani, V. Barone, B. Mennucci, G. A. Petersson, H. Nakatsuji, M. Caricato, X. Li, H. P. Hratchian, A. F. Izmaylov, J. Bloino, G. Zheng, J. L. Sonnenberg, M. Hada, M. Ehara, K. Toyota, R. Fukuda, J. Hasegawa, M. Ishida, T. Nakajima, Y. Honda, O. Kitao, H. Nakai, T. Vreven, J. A. Montgomery, Jr., J. E. Peralta, F. Ogliaro, M. Bearpark, J. J. Heyd, E. Brothers, K. N. Kudin, V. N. Staroverov, T. Keith, R. Kobayashi, J. Normand, K. Raghavachari, A. Rendell, J. C. Burant, S. S. Iyengar, J. Tomasi, M. Cossi, N. Rega, J. M. Millam, M. Klene, J. E. Knox, J. B. Cross, V. Bakken, C. Adamo, J. Jaramillo, R.

Gomperts, R. E. Stratmann, O. Yazyev, A. J. Austin, R. Cammi, C. Pomelli, J. W. Ochterski, R. L. Martin, K. Morokuma, V. G. Zakrzewski, G. A. Voth, P. Salvador, J. J. Dannenberg, S. Dapprich, A. D. Daniels, O. Farkas, J. B. Foresman, J. V. Ortiz, J. Cioslowski, D. J. Fox, Gaussian, Inc., Wallingford CT, 2010.

[2] a) T. Kubo, *Chem. Lett.* 2015, **44**, 111–122; b) S. Das, J. Wu, in *Polycyclic arenes and heteroarenes: synthesis, properties, and applications*, (Ed.: Q. Miao), John Wiley & Sons Inc., 2015, 3.

[3] K. Yamaguchi, *Chem. Phys. Lett.* 1975, **33**, 330–335.

[4] Z. Chen, C. S. Wannere, C. Corminboeuf, R. Puchta, P. v. R. Schleyer, *Chem. Rev*. 2005, **105**, 3842–3888.

[5] N. M. O'boyle, A. L. Tenderholt, K. M. Langner, *J. Comput. Chem*. 2008, **29**, 839–845.

[6] P. Hu, S. Lee, T. S. Herng, N. Aratani, T. P. Gonçalves, Q. Qi, X. Shi, H. Yamada, K. Huang, J. Ding, D. Kim, J. Wu, *J. Am. Chem. Soc.* 2016, **138**, 1065–1077.

[7] Bruker, SAINT V8.38A, (Bruker AXS Inc., 2017)

[8] O. V. Dolomanov, L. J. Bourhis, R. J. Gildea, J. A. Howard, H. Puschmann, *J. Appl. Crystallogr.* 2009, **42**, 339–341.

[9] L. J. Bourhis, O. V. Dolomanov, R. J. Gildea, J. A. Howard, H. Puschmann, *Acta Crystallogr. A*. 2015, **71**, 59–75.

[10] G. M. Sheldrick, *Acta Crystallogr. C Struct. Chem*. 2015, **71**, 3–8.
